# Abundant CpG-sequences in human genomes inhibit KIR3DL2-expressing NK cells

**DOI:** 10.7717/peerj.12258

**Published:** 2021-11-05

**Authors:** Jason Pugh, Lisbeth Guethlein, Peter Parham

**Affiliations:** Departments of Structural Biology and Microbiology & Immunology, Stanford University School of Medicine, Stanford, CA, United States

**Keywords:** HLA, KIR, NK cells, KIR3DL2, CpG-DNA, PAMP

## Abstract

Killer Immunoglobulin-like Receptors (KIR) comprise a diverse, highly polymorphic family of cell-surface glycoproteins that are principally expressed by Natural Killer (NK) cells. These innate immune lymphocytes fulfill vital functions in human reproduction and immune responses to viral infection. KIR3DL2 is an inhibitory NK cell receptor that recognizes a common epitope of the HLA-A3 and HLA-A11 class I glycoproteins of the major histocompatibility complex. KIR3DL2 also binds exogenous DNA containing the CpG motif. This interaction causes internalization of the KIR-DNA. Exogenous CpG-DNA typically activates NK cells, but the specificity of KIR3DL2-DNA binding and internalization is unclear. We hypothesized that KIR3DL2 binds exogenous DNA in a sequence-specific manner that differentiates pathogen DNA from self-DNA. In testing this hypothesis, we surveyed octameric CpG-DNA sequences in the human genome, and in reference genomes of all bacteria, fungi, viruses, and parasites, with focus on medically relevant species. Among all pathogens, the nucleotides flanking CpG motifs in the genomes of parasitic worms that infect humans are most divergent from those in the human genome. We cultured KIR3DL2^+^NKL cells with the commonest CpG-DNA sequences in either human or pathogen genomes. DNA uptake was negatively correlated with the most common CpG-DNA sequences in the human genome. These CpG-DNA sequences induced inhibitory signaling in KIR3DL2^+^NKL cells. In contrast, KIR3DL2^+^NKL cells lysed more malignant targets and produced more IFNγ after culture with CpG-DNA sequences prevalent in parasitic worms. By applying functional immunology to evolutionary genomics, we conclude that KIR3DL2 allows NK cells to differentiate self-DNA from pathogen DNA.

## Introduction

The immune system uses receptors to recognize pathogen-associated molecular patterns (PAMPs) and distinguish them from self. One such PAMP is exogenous DNA that contains a Cytosine followed by a Guanine in the 5′ to 3′ orientation, in which the Cytosine is unmethylated (CpG motif) (Gautier, Bünemann & Grotjahn, 1977; Krieg et al., 1995). The CpG motif signifies pathogenic DNA, in part because it occurs infrequently in mammalian genomes (Bird, 1980).

Natural Killer (NK) cells are innate immune cells that orchestrate the early immune response by secreting cytokines and lysing infected or malignant cells (Piccioli et al., 2002; Andrews et al., 2010). NK cells also sense exogenous DNA (Yamamoto et al., 1992). Despite these general and fundamental functions, most inherited deficiencies of NK cells are associated with uncontrolled outbreaks of herpesvirus infections (Orange, 2002). Further supporting a principle role for NK cells in defense against herpes viruses, is the presence in mice of receptors that bind specifically to herpes virus proteins (Arase et al., 2002; Min-Oo & Lanier, 2014).

NK cells survey cell surfaces using both activating and inhibitory receptors. This enables NK cells to preserve healthy cells, while killing cells that are infected or malignant. Of particular importance for these mechanisms are the family of Killer-Immunoglobulin-like Receptors (KIR), which recognize polymorphic epitopes of HLA class I (Parham & Moffett, 2013).

KIR3DL2 is an inhibitory receptor (Uhrberg, Parham & Wernet, 2002) that recognizes an epitope shared by the HLA-A3 and HLA-A11 allotypes of HLA-A (Döhring et al., 1996; Pende et al., 1996). When KIR3DL2 binds to its target epitope on a healthy cell, the ITIMs of KIR3DL2 become phosphorylated and recruit the inhibitory SHP1 and SHP2 phosphatases to the signaling complex. On activation of SHP1 and SHP2, the lytic and inflammatory functions of the NK cell are prevented from attacking healthy cells (Veillette, Latour & Davidson, 2002; Purdy & Campbell, 2009). In contrast, infected or malignant cells can have reduced expression of HLA-A that facilitates NK cell activation and target cell killing. In this way, KIR3DL2^+^ NK cells respond with a strong lytic and inflammatory response toward cells with reduced surface expression of self HLA-A.

Exogenous DNA can also be bound and internalized by KIR3DL2. When KIR3DL2^+^ NK cells are stimulated with IL-12 or IL-8, DNA is internalized by KIR3DL2 and is passed onto TLR-9 in endosomes (Sivori et al., 2010). Studies of NK cell responses to exogenous DNA have tended to focus on a small number of CpG-DNA sequences that have strong immune activating capacity (Dalpke et al., 2002; Hartmann et al., 2003).

We examined the genomes of all known human pathogens and determined the presence and frequency of nucleotides flanking their CpG motifs. Most similar to CpG-DNA sequences in the human genome were those in herpes viruses, while least similar were those in parasitic worms. Based on genomic frequencies among pathogens, we used a transfectant of the NKL cell line that expresses KIR3DL2 to examine the uptake and immune modulation of various CpG-DNA sequences. Those sequences more commonly found in the human genome were taken up less, and caused inhibitory signaling in KIR3DL2^+^NKL cells. In contrast, CpG-DNA sequences that are abundant in parasitic worm genomes enhanced the immune functions of KIR3DL2^+^NKL cells. Previous studies concentrated on the stimulatory effects of unmethylated CpG-DNA on NK cells. The investigation described here used comparative genomics to show that KIR3DL2 discriminates between self and non-self DNA using nucleotides that flank the CpG motif, thereby altering the immune response.

## Materials & Methods

### Cell lines and cell culture

All human cells used in this study were previously established lines derived from de-identified individuals, and as such are exempt from IRB approval. 721.221 cells were generated as described previously (Sanjanwala et al., 2008). Cells were maintained in complete Roswell Park Memorial Institute 1640 medium (Corning Inc., Corning, NY, USA) supplemented with 2mM L-glutamine (Thermo Fisher Scientific, Waltham, MA, USA), 100 U/ml penicillin and streptomycin (Thermo Fisher, Waltham, MA, USA), and 15% Fetal Bovine Serum (Corning, Corning, NY, USA). Hereafter, this medium is referred to as RPMI-15%C medium.

NKL lines were generated as described previously (Norman et al., 2009). Briefly, KIR3DL2 cDNA was isolated from PBMC and cloned into the pBMN retroviral vector. Retrovirus was generated by transfection of pBMN into Phi-NX cells. Supernatants containing retrovirus were used to infect NKL cells. Retrovirally infected NKL cells were sorted based on KIR expression using a FACSvantage cell sorter (BD biosciences, San Jose, CA, USA). NKL cells were maintained in RPMI-15%C medium supplemented with 250 U/ml recombinant human Interleuikin-2 (IL-2). IL-2 was obtained from either Gibco (Thermo Fisher, Waltham, MA, USA) or from Dr. Maurice Gately (Hoffmann-La Roche Inc., Basel, Switzerland), through the National Institutes of Health AIDS Reagent Program, Division of AIDS, National Institute of Allergy and Infectious Diseases, National Institutes of Health. For experiments using IL-12, NKL cells were cultured for 48 hours in RPMI-15%C medium supplemented with 500 U/ml IL-2 and 50 ng/ml recombinant human IL-12 (eBioscience/Thermo Fisher Scientific, Waltham, MA, USA).

### DNA oligonucleotides

DNA oligonucleotides (ODN) were synthesized and purified by the Stanford Protein and Nucleic Acid Facility (Stanford PAN Facility, Stanford, CA, USA). Lyophilized ODN were resuspended to 100 μM in 1× Dulbecco’s phosphate buffered saline (DPBS, Gibco/Thermo Fisher, Waltham, MA, USA). Oligo suspensions were stored at 4 °C in the dark, to avoid freeze-thaw cycles.

### Culture of ODN with cells

Two 96-well U-bottom, polystyrene, tissue culture-treated plates (Fisherbrand, Thermo Fisher, Waltham, MA, USA) were used to prepare these cultures. To plate 1 was added 10 μl of 100 μM oligo or 3′FITC oligo in 1×DPBS. To plate 2 was added approximately 20,000 cells in RPMI-20%C medium with 250 U/ml IL-2. Plate 2 was spun at 700×*g* at room temperature for 3 min. Excess medium was removed from plate 2, leaving approximately 20 ul of cell suspension remaining in each well. A total of 10 μl of suspended cells from plate 2 were transferred to plate 1. By transferring cell suspensions to oligo suspensions using a multi-channel pipet, deviations in the time of interaction were minimized. Cells were mixed with ODN by pipetting gently several times. Cells were then cultured at 37 °C in 5% CO_2_ in the dark for the durations indicated in each experiment.

### Live/Dead staining of cells

Prior to Live/dead staining, cells were washed three times with ice-cold 1×DPBS by spinning at 700× *g* in a 4 °C centrifuge. Live/Dead stain was prepared by mixing 2 μl of fixable far-red dead cell stain stock solution (Invitrogen/Thermo Fisher, Waltham, MA, USA) with one ml 1×DPBS. Cells were suspended in 50 μl of Live/Dead stain and then incubated at 4 °C for 30 min in the dark. Excess Live/Dead stain was removed by washing cells once in 1xDPSB, and then once in FACs buffer consisting of 1×DPBS and 5%FBS.

### DNA uptake assays

For assays using FITC-labeled ODN, 10,000 cells were cultured with ODN at 37 °C in 5% CO_2_ for the durations indicated. Cultures occurred in 96-well U-bottom plates. Cells were then stained with Live/Dead dye, and assayed by flow cytometry.

DNA uptake assays using intercalating DNA dye were performed the same as assays using FITC-labeled DNA (See *Culture of ODN with cells* above), except for the following. Prior to mixing cells with ODN, Gelred dye (Biotium, Inc., Fremont, CA, USA) was diluted 1:10,000 in 1×DPBS. A total of 10 μl of the dye solution was then added to each well of ODN in plate 1. Plate 1 was then incubated for 10 min at room temperature in the dark, in order to allow the dye to bind DNA. Then, the cells were added from plate 2, as described above. Medium was then supplemented with additional FBS to a final concentration of 10%.

### Functional assays

For lysis assays, NKL cells were cultured with unlabeled ODN or without ODN for four hours as described above. Separately, 721.221 cells were labeled with the Cell Trace CFSE cell proliferation kit (Thermo Fisher, Waltham, MA, USA), following the manufacturer’s instructions. To each well of NKL cells was then added 721.221 cells at a 1:10 ratio in 100 μl of RPMI-10%C medium supplemented with 500 U/ml IL-2. Labeled 721.221 cells were also cultured alone, to establish a baseline of survival. Plates were spun at 100×*g* for 2 min at room temperature, to allow NKL cells and target cells to group together on the plate surface. Cells were then co-cultured at 37 °C in 5% CO_2_ for 2.5 h. After co-culture, cells were stained with Live/Dead dye and then assayed by flow cytometry.

A baseline of survival for 721.221 cells in culture was established by gating on the percentage of cells in the living, CFSE^+^ gate among those 721.221 cells cultured alone. The percentage of living CFSE^+^ cells in each co-culture was then divided by this baseline percentage, creating an adjusted percentage of target cells that escaped lysis. The adjusted percentage for each co-culture containing NKL cells treated with DNA was then divided by the adjusted percentage for the co-culture containing NKL cells that were not treated with DNA. The resulting ratio describes the enhancement or diminishment of lysis by NKL cells due to exposure to exogenous DNA.

Assays measuring IFNγ began with the DNA culture with NKL, followed by co-culture with 721.221 cells. However, 721.221 cells were not labeled with CFSE for IFNγ assays. After one hour of co-culture with target cells, Brefeldin A (Sigma-Aldrich, St. Louis, MO, USA) was added to each well to a final concentration of five μg/ml. Co-cultures continued for five more hours. After Live/Dead staining, co-cultures were fixed with 100 μl BD cytofix fixative (BD Biosciences, Franklin Lakes, NJ, USA) for 15 min at 4 °C. A total of 100 μl of BD cytoperm wash (BD Biosciences, Franklin Lakes, NJ, USA) was then added to each well. Cells were washed two more times with BD cytoperm wash, using centrifugations of 3 min at 700×*g* in 4 °C between each wash. To make “perm-plus” solution, one ml of 10× BD cytoperm concentrate was added to one ml Dimethyl sulfoxide (EMD Millipore, Burlington, MA, USA), and the solution was diluted to 10 ml with H_2_O. This perm-plus solution permeates intracellular membranes. A total of 200 μl of perm-plus solution was then added to each well, and the cells were incubated for 20 min at 4 °C. Cells were then washed three times with BD perm wash, fixed again with BD cytofix for 15 min, and washed twice more with BD perm wash. The second fixation treatment ensures fixation of intracellular compartments. Cells were then stained with anti-IFNγ antibody in 50 μl of BD cytoperm solution.

The percentage of living, IFNγ^+^ cells in each co-culture containing NKL cells treated with DNA was then divided by the percentage of living, IFNγ^+^ cells in co-cultures containing NKL cells treated without DNA. The resulting ratio describes the enhancement or diminishment of IFNγ due to exogenous DNA.

### Phospho-flow

Cells were cultured with ODN for 4 h. After culture, cells were washed three times with 1×DPBS to remove excess DNA. After pelleting, cells were re-suspended in 200 μl of 1× BD Phosflow lyse/fix buffer (BD Biosciences, Franklin Lakes, NJ, USA), which was pre-warmed to 37 °C. Cells were then incubated at 37 °C for 10 min. After spinning at 700×*g* at room temperature, fixative was removed. Cells were then re-suspended in 200 μl of BD Phosflow perm buffer III (BD Biosciences, Franklin Lakes, NJ, USA), which was pre-chilled to −20 °C. Cells were permeabilized at 4 °C for 20 min. After two washes with FACs buffer, staining with anti-phosflow antibodies (5 μl/test) occurred in 50 μl of FACs buffer at room temperature in the dark for 1 h. Cells were washed twice more with FACs buffer to remove excess antibody, and then analyzed by flow cytometry. Live cells were discriminated from dead in phosflow experiments using FSC-A and SSC-A.

### Trypsin digestion of surface KIR3DL2

After culture with ODN, cells were pelleted at room temperature for 3 min at 700×*g*. Excess medium was removed and cells were re-suspended in 100 μl Trypsin-EDTA 0.05% with phenol red (Thermo Fisher, Waltham, MA, USA). After incubation at 37 °C for 10 min, cells were washed three times with ice-cold 1×DPBS, stained with Live/dead stain, and analyzed by flow cytometry.

### Flow cytometry

Fluorescently-labeled monoclonal antibodies were used to stain cells prior to flow cytometry. These included anti-CD19 (clone HIB19, Biolegend), anti-KIR3DL1 (clone DX9, Biolegend, San Diego, CA, USA), anti-SHP1-Y536 (clone Shp1Y536-2A7, Thermo Fisher, Waltham, MA, USA), anti-SHP2-Y580 (clone Shp2Y580-4A2, Thermo Fisher, Waltham, MA, USA), and anti-IFNγ (clone B27, Biolegend, San Diego, CA, USA). The monoclonal antibody clone DX31, a kind gift from Louis Lanier, was used to stain cells for the presence of KIR3DL2. DX31 was visualized with polyclonal PE conjugated Goat anti-mouse Ig antibody (BD biosciences, Franklin Lakes, NJ, USA). Flow cytometry was performed on a BD Accuri flow cytometer, or a BD Aria II flow cytometer (BD Biosciences, Franklin Lakes, NJ, USA) maintained at the Stanford Shared FACS Facility, Stanford, CA. Data were analyzed using FlowJo Ver. 10.6.1 (FlowJo, LLC Ashland, OR, USA).

### Genomic datasets

Genomes were acquired from the NCBI database (https://www.ncbi.nlm.nih.gov/) or Wormbase (https://wormbase.org//#012-34-5). Dataset B contained the reference genomes of all available bacteria, and was downloaded on 2/12/2020. Dataset F contained the reference genomes of all available fungi, and was downloaded on 1/2/20. Species in dataset P were downloaded on 1/3/2020. Dataset V included all virus reference genomes, and was downloaded on 11/24/2019. Species in dataset HV were acquired by selecting “Human” as the host species on the viral genomes browser at https://www.ncbi.nlm.nih.gov/genomes/GenomesGroup.cgi?taxid=10239. Subsets of data containing ssDNA viruses infecting vertebrates were acquired in the same way. The resulting datasets were manually screened to exclude RNA viruses by taxonomy. Herpes virus and ssDNA virus subsets were isolated manually from the larger HV dataset. Plasmids referenced to a species were included in the dataset containing that species. For species-level analysis, occurrences of CpG^+^ octamers from all datasets pertaining to that species were summed. A list of the fasta files used in this study can be found in Data S5.

### Computational analysis

Supplemental data 6 is a repository containing examples of the executable programs used in this study, and can be found at https://github.com/jpugh-sudo/Pugh_SF_6. Programs were compiled and executed using Perl v.5.26.1 built for x86_64-linux-gnu-thread-multi in a Linux environment 4.15.0-132-generic GNU/Linux. Workload was managed using Slurm Ver. 18.08 (SchedMD, LLC, Lehi, UT, USA).

### Statistical analysis

X-Y correlations between genetic frequency and cell-based assays were analyzed by simple linear regression regardless of apparent curvature, so that goodness-of-fit (r^2^) could be compared quantitatively between experimental approaches. For correlations, *p*-values represent the significance of the slope of the linear regression deviating from zero. All *p*-values reported for correlations were Bonferroni-corrected by multiplying the raw *p*-value by the number of correlations performed in the study: 705.

For cell-based assays, the results of replicate experiments were used to establish significance. The results of each experiment were normalized to a control condition, such as a sample without exogenous DNA, so that replicates from multiple experiments could be fairly included in the same analysis. An ordinary 2-way ANOVA was used to establish significance in functional or signaling outcomes involving CpG octamers, with species-of-origin as the reported factor. 2-way ANOVA was chosen so that significant differences between species-of-origin could be calculated independent of the distribution of first, second, and third-ranked octamers within a species.

Statistical significance was assessed using Prism Ver. 6 (Graphpad software, San Diego, CA, USA). For Bonferroni corrections, actual *p*-values were calculated and adjusted in Microsoft Excel for Mac version 14.7.7 (Microsoft Inc., Redmond, WA, USA).

## Results

### The footprint of KIR3DL2 binding to CpG-DNA extends four bases upstream of the CpG motif

We hypothesized that DNA binding by KIR3DL2 evolved as a mechanism that enables NK cells to distinguish non-self, namely pathogens, from self. The hypothesis predicts that KIR-DNA binding is sequence-specific and reflects differences between human and pathogen genomes.

To explore this possibility, we first established basic parameters affecting KIR-DNA binding and uptake. These included the presence, copy number and spacing of the CpG motif within the DNA sequence, in addition to the overall sequence length. We also sought to define experimental conditions favoring *in vitro* uptake of KIR-DNA.

To accomplish these goals, we used a lymphoblastic, human Natural Killer cell line (NKL) that expresses no endogenous KIR genes. We made transfectants of NKL having stable expression of KIR3DL2*001 (the KIR3DL2^+^NKL cell line) or KIR3DL1*001 (the KIR3DL1^+^NKL cell line) (Data S1A).

We obtained a purified DNA oligonucleotide that has the sequence 5′-TCGTCGTTTTGTCGTTTTGTCGTTCATGAA-3′ (Seq1). This sequence is based on that used to stimulate NK cells in many previous studies (Hartmann et al., 2003). We made an adduct of Seq1 that has a terminal 3′ nucleotide attached to a fluorescein molecule (FITC), in order to track cellular binding and uptake of the oligonucleotide. FITC was chosen specifically because cyanine dyes can bias the results of DNA uptake experiments (Lacroix et al., 2019). CpG^+^ oligonucleotides (ODN) with 3′ FITC will be called CpG-ODN-3′FITC hereafter.

KIR3DL2 has been reported to bind and internalize DNA more effectively than other inhibitory KIR (Sivori et al., 2010). We verified this observation by separately culturing ten thousand KIR3DL2^+^NKL, KIR3DL1^+^NKL or KIR^-^NKL cells with 20 μM Seq1, in a cell culture-treated 96-well U-bottom plate. Cultures lasted 20 min in 20 μl of RPMI medium with 5% FBS and 500 U/ml IL-2 at 37 °C in 5% CO_2_. After thoroughly washing the cells with 1×DPBS, they were stained with Live/Dead dye. The geometric mean fluorescence intensity (gMFI) in the FITC channel on living cells was then determined by flow cytometry. Consistent with the previous studies, gMFI in the FITC channel was ten-fold greater for KIR3DL2^+^NKL cells than for KIR3DL1^+^NKL or KIR^−^NKL cells (Data S1B).

This method of culturing NKL with exogenous FITC-labeled DNA effectively measures the combined binding and internalization of exogenous DNA by the cultured NKL cells. This technique was used with variations for the experiments reported in this section of results.

To establish the saturating concentration of CpG-ODN for KIR^+^NKLs, we cultured KIR3DL2^+^NKL and KIR3DL1^+^NKL cells separately at five concentrations of Seq1: 2.5, 5, 25, 50 and 75 μM. For both KIR3DL2^+^NKL and KIR3DL1^+^NKL cells, the FITC gMFI obtained after culture with 50 μM Seq1 was similar to that obtained with 75 μM Seq1 (Data S1C). A DNA concentration of 50 μM was thus sufficient to saturate the uptake of DNA by KIR^+^NKLs.

We next determined whether the observed uptake of DNA by KIR3DL2^+^NKL cells was an active process, or the result of passive adhesion. We reasoned that an active process would require physiological temperature, as well as cell motility. KIR3DL2^+^NKL cells were cultured with Seq1 for 20 min in polystyrene plates that had either been treated for cell culture, or not, at either 4 °C or 37 °C. The KIR3DL2^+^NKL cells in the cell culture-treated plates at 37 °C had a mean uptake of CpG^+^DNA that was 31% greater than that of KIR3DL2^+^NKL cells cultured at 4°C or cultured in untreated plates (Data S1D). This result suggests that KIR3DL2^+^NKL cells acquire exogenous CpG^+^DNA through an active mechanism, as well as by passive adhesion.

We tested the importance of the CpG motif for the uptake of DNA by comparing the uptake of two 36-mers by KIR3DL2^+^NKL cells. One 36-mer contained a central CpG motif flanked by thymine (CpG-ODN-3′FITC) and the other contained a GpC motif (GpC-ODN-3′FITC). Generally, poly(T) was chosen over poly(A) to flank the central motif in our experiments, to be consistent with the reported requirements of TLR-9 (Pohar et al., 2015). The KIR3DL2^+^NKL cells cultured with CpG-ODN-3′FITC achieved a gMFI that was 2.6 fold that obtained with GpC-ODN-3′FITC (Data S1E). This result demonstrates that the CpG motif is advantageous but not essential for the uptake of exogenous DNA by KIR3DL2^+^NKL cells.

We then compared the uptake of single-stranded (ss) DNA and double-stranded (ds) DNA by KIR3DL2^+^NKL cells. CpG-ODN-3′FITC’s of ten different lengths were made with a central CpG motif flanked by thymines (Data S2A). Each CpG-ODN-3’FITC was bound to its unlabeled reverse-complement CpG-ODN, thereby producing ds-CpG-ODN-3′FITC that would deliver the same FITC signal per molecule as the ss-CpG-ODN-3′FITC. Both ss and ds CpG-ODN-3′FITC were cultured with KIR3DL2^+^NKL cells. On average, the FITC gMFI obtained by KIR3DL2^+^NKL cells cultured with ssDNA was 1.86 fold greater than that obtained by the cells cultured with dsDNA (student’s t test, *p* < 0.001, Data S1F).

We determined the kinetics of DNA uptake by culturing KIR3DL2^+^NKL cells with Seq1 for time periods ranging from 1 min to 1 h, and in further experiments for times ranging from 1 to 5 h. The greatest increases in DNA uptake, 5.9-fold, were achieved by KIR3DL2^+^NKL cells cultured with DNA for 30 min to 1 h (Data S1G). These results point to a sigmoidal curve for DNA uptake over time.

**Figure 1:**
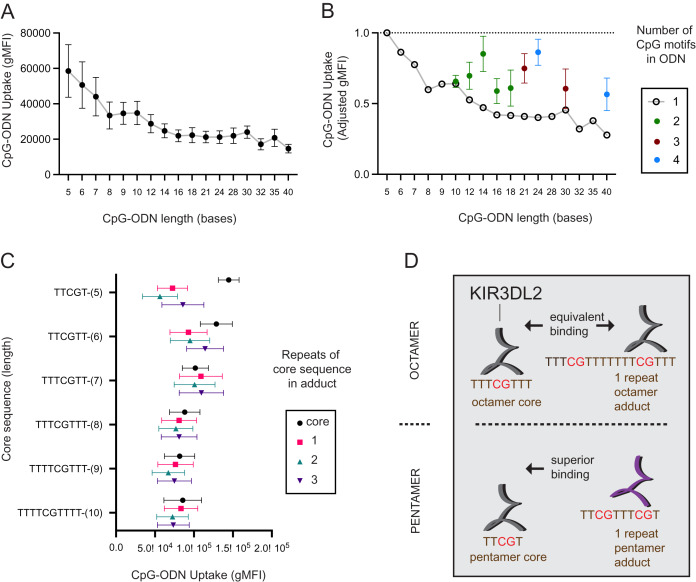
KIR3DL2^+^NKL cells preferentially take up short oligonucleotides having CpG motifs spaced three or more bases apart. (A) FITC-conjugated oligonucleotides were synthesized to have a CpG motif at the center, flanked by a variable number of thymines to give oligonucleotides of different length. Oligonucleotides were individually cultured with KIR3DL2^+^NKL cells. The y-axis gives the geometric mean fluorescence intensity (gMFI) of the live cells, as determined by flow cytometry. Replicate cultures were incubated for 4, 8, 12, 18 or 24 h. The mean oligonucleotide binding for the five cultures is plotted. (B) Comparison of KIR3DL2^+^NKL cell uptake of FITC-oligonucloeotides containing one CpG motif (open circles), or oligonucleotides of the same length containing >1 CpG motif (filled circles). Shown are the results of four replicate experiments. The FITC signal is normalized to that of KIR3DL2^+^NKL cells cultured with the FITC-conjugated pentamer TCGTT. (C) FITC-labeled oligonucleotide adducts were designed to have repeating core CpG^+^ sequences of different lengths, shown on the y-axis. Adducts are composed of the core sequence repeated 1, 2, or 3 times consecutively. Core oligonucleotides and adducts were individually cultured with KIR3DL2^+^NKL cells. Each data point is the average for the 4, 12 and 24 h cultures. Shown is the gMFI in the FITC channel. (D) A schematic summarizing the results of panel C. In an adduct composed of repeated octamers, each containing one CpG motif, the CpG motifs are sufficiently far apart that KIR3DL2^+^NKL cells take up the adduct and the octamer to nearly the same extent. So in this instance, having multiple CpG motifs does not increase the uptake. When CpG motifs are too close together, as in an adduct composed of repeated pentamers, then KIR3DL2 binding is diminished. Consequently, no oligonucleotide having multiple CpG motifs exceeds the uptake of the core CpG^+^ pentamer.

To see how the length of a DNA molecule affects its uptake by KIR3DL2^+^NKL cells, seventeen CpG-ODN-3′FITC were made that ranged in length (L) from 5–40 nucleotides (L = 5 - L = 40). L = 5 reflects the minimum length CpG-ODN known to provide immunostimulation (Fearon et al., 2003). Each sequence comprised a central CpG motif flanked by thymidines (Data S2A). KIR3DL2^+^NKL and KIR^−^NKL cells were cultured separately with each of the seventeen CpG-ODN-3′FITC for 4, 8, 12, 18, and 24 hours, and then assayed by flow cytometry. For each CpG-ODN-3′FITC, a mean gMFI for the five time points was calculated. Overall, the mean gMFI obtained by the KIR3DL2^+^NKL cells decreased with increasing length of the CpG-ODN (Fig. 1A). The mean gMFI obtained by KIR3DL2^+^NKL cells cultured with the 40mer oligonucleotide was 28% of cells cultured with the pentamer (Fig. 1A). The variation in average gMFI decreased as the oligonucleotide’s length increased. These results show how KIR3DL2^+^NKL cells prefer short DNA molecules, a preference that becomes more pronounced with increasing time of culture with exogenous DNA.

We also examined how the number of CpG motifs in a DNA molecule affects its uptake by KIR3DL2^+^NKL cells. Five CpG-ODN-3′FITC were made to range from 10 to 18 nucleotides in length (L = 10–L = 18). These five CpG-ODN-3′FITC had two CpG motifs (2xCpG) flanked by thymines. Similarly made were two 3xCpG-ODN-3′FITC (L = 21, L = 30) containing three CpG motifs and two 4xCpG-ODN-3′FITC (L = 24, L = 40) containing four CpG motifs (Data S2B). KIR3DL2^+^NKL cells were cultured separately with each of the 2x, 3x, or 4xCpG-ODN-3′FITC, as well as a length-matched 1xCpG-ODN-3′FITC.

For most of the oligonucleotides tested, having more than one CpG motif increased the uptake of the CpG-ODN-3′FITC by KIR3DL2^+^NKL cells (Fig. 1B). The average increase in the uptake of CpG-ODN having one additional CpG (2xCpG) ranged from 60% (L = 12) to 136% (L = 14). For CpG-ODN having two or three additional CpG motifs the uptake ranged from 89% (L = 30) to 175% (L = 40). Thus the uptake of CpG-ODN by KIR3DL2^+^NKL cells does not increase linearly with the number of CpG motifs.

The distance in nucleotides between successive CpG motifs in a genome is known as the CpG interdistance (CpG-ID). To test the effect of CpG-ID on the uptake of DNA by KIR3DL2^+^NKL cells, we manufactured eight 2xCpG-ODN-3′FITC. Each of the eight 2xCpG-ODN-3′FITC was 14 bases long, but they differed in CpG-ID, which ranged from one to eight nucleotides (Data S2C). KIR3DL2^+^NKL cells were cultured with each 2xCpG-ODN-3′FITC, and then analyzed by flow cytometry. The 2xCpG-ODN-3′FITC having a seven nucleotide CpG-ID was taken up by KIR3DL2^+^NKL cells to a greater extent than the seven other oligonucleotides (Data S1D).

Thus far our data shows that no 2x, 3x, or 4x CpG-ODN-3′FITC exceeded the uptake obtained by the shortest 1xCpG-ODN-3′FITC that is five nucleotides long (L = 5, Fig. 1B). We also found that increasing CpG-ID generally increased oligonucleotide uptake by KIR3DL2^+^NKL cells. Together, these observations suggest that longer oligonucleotides, having multiple CpG motifs, are taken up by KIR3DL2^+^NKL cells as if they are a combination of shorter oligonucleotides with single CpG motifs.

To test this hypothesis, we designed a series of 2x, 3x, or 4xCpG-ODN-3′FITC in which a core 1xCpG sequence is consecutively repeated: one, two or three times. For example, the 1xCpG-ODN pentamer TCGTT was duplicated to give the 2xCpG-ODN adduct TCGTTTCGTT. Another TCGTT was placed after this sequence to give the 3xCpG-ODN adduct TCGTTTCGTTTCGTT, and so forth. Overall, CpG-ODN-3′FITC were made to have core 1xCpG sequences of 5–10 nucleotides, repeated 1–3 times consecutively (Data S2E). Each of these twenty-four CpG-ODN-3′FITC were cultured with KIR3DL2^+^NKL cells and then analyzed by flow cytometry. For adducts having core sequences of seven or more nucleotides, repetition of the core sequence had no significant effect on the uptake of the nucleotide by KIR3DL2^+^NKL cells (Fig. 1C). In contrast, adducts composed of repeated pentameric sequences were taken up only 50% of the extent of a single pentamer (Fig 1C and 1D).

**Figure 2:**
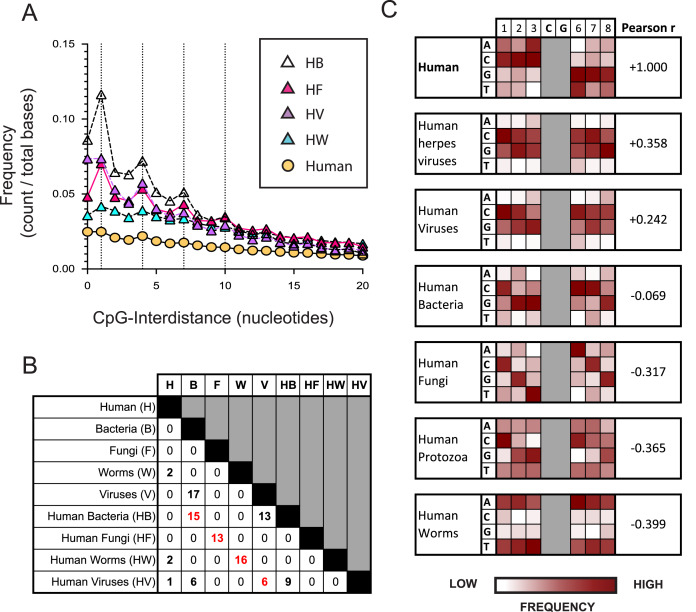
The nucleotide sequences flanking CpG motifs in parasitic worm genomes differ more from those in the human genome than the corresponding CpG motifs of bacteria, viruses and fungi. (A) The distance between one CpG motif and the next in genomic datasets comprising different evolutionary groups. Datasets include the human genome (H) and genomes of the bacteria (HB), fungi (HF), viruses (HV) and parasitic worms (HW) that infect humans. Dotted vertical lines highlight the frequency of CpG interdistances of 1, 4, 7, and 10 bases. (B) The number of CpG^+^ octamers that are common to the twenty most frequent CpG^+^ octamers in each dataset. (C) Distribution of nucleotides in octamer sequences with a central CpG site in each genomic dataset. The distribution of CpG^+^ octamers in the human genome is compared to their distribution in each non-human dataset using the Pearson correlation coefficient.

These results show that a DNA sequence containing multiple CpG motifs is taken up by KIR3DL2 to the same extent as the 1xCpG sequences forming that sequence, providing the CpG motifs are separated by five or more nucleotides (compare repeats of hexamers and heptamers, Fig. 1C). The correct interpretation of Fig. 2B is not that additional CpG motifs increase oligonucleotide uptake, but that KIR3DL2 prefers shorter sequences, and adding CpG motifs with the correct spacing allows KIR3DL2 to take up a long oligonucleotide to the same extent as a short one (Fig. 1D).

### The nucleotides flanking CpG motifs in parasite genomes differ most from those in human genomes

Infectious diseases of humans are mainly caused by four groups of pathogen: bacteria, viruses, fungi and parasitic worms. To assess their differences and similarities, genomic datasets were compiled for the reference genomes of all of the bacteria (B), viruses (V), fungi (F) and worms (W).

From these four datasets, the genomes of species most relevant to human disease were collected. In a first phase, species belonging to the same genus as a known human pathogen were identified and their genome sequences compiled. In a second phase, all species having an entry in the NIH Medgen database were identified and their genome sequences compiled (see “Materials and Methods”). This process yielded four genomic datasets: bacterial pathogens that infect humans (HB), viral pathogens that infect humans (HV), parasitic worms that infect humans (HW) and fungal pathogens that infect humans (HF). A complete list of the species included in each dataset is given in Data S5.

We examined how the pattern of KIR3DL2 binding to DNA relates to the distribution of CpG-ID’s present within pathogen genomes. An algorithm was developed to calculate the lengths of all CpG-IDs in a genome and was applied to the eight pathogen datasets and the consensus human genome. The frequency of each CpG-ID value was divided by the size of the genomic dataset, to give a relative frequency for each CpG-ID.

In all genomes analyzed, including non-pathogenic microorganisms and pathogenic microorganisms that infect humans, the CpG-ID’s 1, 4, 7, and 10 were more frequent than neighboring CpG-ID’s (Fig. 2A and Data S3A). The pattern 1, 4, 7, etc. can be represented by the arithmetic formula 3x-2. This 3x-2 formula resembles the pattern of DNA binding by KIR3DL2^+^NKL cells with regard to CpG-ID (compare Fig. 2A and Data S2E).

We next chose a symmetrical unit of CpG^+^ DNA with which to survey the genomic datasets. Sequences of eight nucleotides with a central CpG^+^ site, xxxCGxxx, hereafter called CpG^+^ octomers, were counted in each of our eight genomic datasets and in the human genome.

For each dataset, the twenty most frequent CpG^+^ octamers were compiled. The top-20 list for each dataset was compared for similarity with the top-20 list of every other dataset (Fig. 2B). Not one CpG^+^ octamer among the 4,096 possibilities was present in all eight top-20 lists (Data S3B).

For each genomic dataset, we determined the relative frequency of each possible nucleotide (A,G,C or T) in each of the six positions flanking the central CpG motif in a CpG^+^ octamer (*1-2-3*-C-G-*6-7-8*, Fig. 2C). Using the Pearson correlation coefficient (Pearson r), we quantified the similarity of nucleotides in CpG^+^ octamers between the human genome and each set of pathogenic genomes. In this context, a Pearson r closer to +1 represents greater similarity to the human genome, whereas those closer to −1 are less similar.

Most similar to the distribution of nucleotides flanking CpG motifs in the human genome, is that of the viruses that infect humans (Pearson r = 0.3215). Because an important immunological role for NK cells is the control of herpesviruses, we analyzed a further dataset consisting only of human herpesvirus genomes. The distribution of nucleotides flanking CpG motifs in human herpesviruses was found to be more similar to the human genome than that of all human viruses (Pearson r = 0.3580, Fig. 2C).

**Figure 3:**
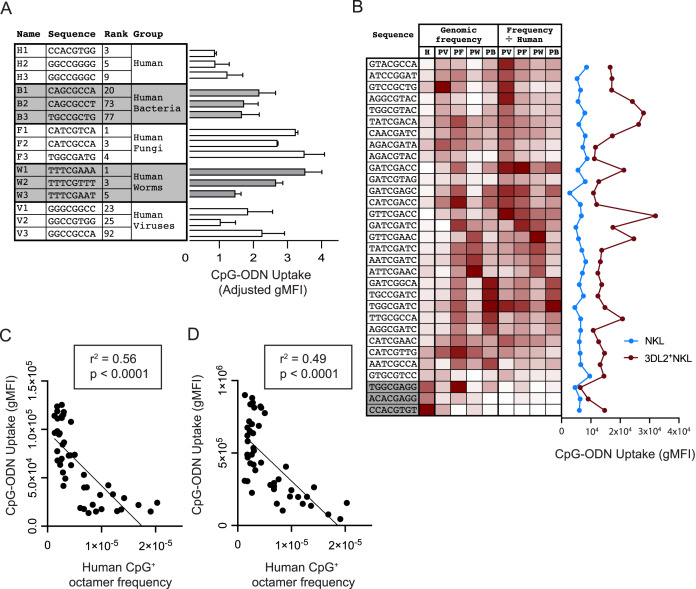
Common CpG^+^ octamers in the human genome are poorly taken up by KIR3DL2^+^NKL cells. (A, Left) From analysis of the human genome, and the genomes of the four groups of human pathogens, we identified frequent octamer sequences that contain one CpG motif and are not included in the 20 most frequent octamer sequences of any other group. A simple rank order was derived, ranging from the most frequent (1) to the least frequent (4,096). Shown are the three most frequent octamers in each pathogen group that fit these criteria, and their rank. (A, Right) The uptake by KIR3DL2^+^NKL cells of the CpG^+^ octamers listed on the left. Shown is the average of two experimental approaches: (1) gMFI signal of KIR3DL2^+^NKL cells cultured with FITC-labeled oligonucleotides, and (2) the fluorescent signal of KIR3DL2^+^NKL cells cultured with unlabeled oligonucleotides and an intercalating DNA dye. The uptake of each pathogen-derived octamer is normalized to the average of the three octamers representing the human genome. (B, Left) Thirty-one CpG^+^ octamers and a heatmap of their frequencies in the human genome (H) and in genomes of the bacteria (HB), fungi (HF), viruses (HV) and parasitic worms (HW) that infect humans. The rightmost four columns show the pathogen genome frequency divided by the human genome frequency. (B, Right) Uptake of the CpG^+^ octamers listed on the left by either NKL cells or KIR3DL2^+^NKL cells. Shown is the gMFI of FITC-labeled CpG^+^ octamers. (C) Correlation of the relative frequency of 48 CpG^+^ octamers in the human genome (x-axis) and the gMFI of KIR3DL2^+^NKL cells after 12 h of culture with each FITC-labeled CpG^+^ octamer (y-axis). Shown is a simple linear regression. (D) Correlation of the relative frequency of 48 CpG^+^ octamers in the human genome (x-axis) and the gMFI of KIR3DL2^+^NKL cells after a 12 h culture with each CpG^+^ octamer and an intercalating DNA-dye (y-axis). Shown is a simple linear regression.

Given that KIR3DL2^+^NKL cells prefer exogenous ssDNA over dsDNA, we also compiled genomic datasets consisting only of viruses that use ssDNA during infection or as their primary genetic material (ssDNA-vectors). One dataset included all available ssDNA-vector genomes, a second dataset included ssDNA-vectors that infect vertebrates, and a third dataset comprised only those ssDNA-vectors that infect humans. The dataset of ssDNA-vectors that infect humans contained CpG^+^ octamers that are dissimilar to those of humans (Pearson r = −0.159, Data S3C). However, this dataset was small, and included only 28,097 CpG motifs. As a result, 14% of the possible 4,096 CpG^+^ octamers were present only once, or not at all. The dataset consisting of ssDNA-vectors that infect vertebrates is more robust, containing 88,405 CpG motifs, with all possible CpG octamers represented three or more times. The CpG^+^ octamers in ssDNA-vectors that infect vertebrates are similar to those present in the human genome (Pearson r = +0.294, Data S3C).

CpG^+^ octamers of pathogenic fungi are very different from those of the human genome (Pearson r = −0.3172, Fig. 2C). We also analyzed a dataset comprising the genomes of parasites of the Protista kingdom that infect humans. Collectively, the CpG octomers of protozoa are negatively correlated with those of humans (Pearson r = −0.365, Fig. 2C). Most dissimilar to the nucleotides flanking CpG motifs in the human genome are those of parasitic worms (Pearson r = −0.3985, Fig. 2C).

### Common CpG^+^ sequences in the human genome are poorly taken up by KIR3DL2^+^NKL cells

We examined if KIR3DL2^+^NKL cells prefer to take up the CpG^+^ sequences that predominate in the genomes of human pathogens. For each pathogen group, we chose the three most frequent CpG^+^ octamers. Excluded from selection was any octamer present in the top-20 octamers of more than one pathogen dataset. Because the number of CpG motifs in a DNA molecule can affect its uptake by KIR3DL2^+^NKL cells (Fig. 1B), octamers with more than one CpG motif were also excluded from selection. With these criteria, three CpG^+^ octamers were chosen to represent each of the pathogen datasets and that of the human genome (Fig. 3A). Based on these sequences, fifteen oligonucleotides were synthesized and fluorescein conjugated to give the corresponding CpG-ODN-3′FITC derivatives.

To compare the uptake of the CpG-ODN-3′FITC by KIR3DL2^+^NKL cells, we took two approaches. With the first approach, KIR3DL2^+^NKL cells were cultured separately with each of the fifteen CpG-ODN-3′FITC for 5 h, and then analyzed by flow cytometry. The second approach used 15 CpG-ODN having the same nucleotide sequences as the CpG-ODN-3′FITC, but lacking the fluorescent label. These CpG-ODN were cultured with KIR3DL2^+^NKL cells and GelRed, a DNA dye, and then analyzed by flow cytometry. GelRed is an intercalating DNA dye that also binds to the phosphate backbone of single-stranded DNA. The means of the results obtained with the two approaches were combined for analysis. The CpG-ODNs chosen to represent the human genome were taken up to a lesser extent, 29–66%, than the CpG-ODNs representing pathogen genomes (Fig. 3A).

To characterize the preferences of KIR3DL2^+^NKL cells for particular DNA sequences, we used a set of 28 CpG^+^ octamers that differentially distribute in HV, HB, HF, and HW datasets. Their sequences were selected to represent one or more pathogen groups using a heatmap of the relative frequency of all 4,096 possible CpG^+^ octamers (Fig. 3B). Sequences were excluded from selection if they were among the 15 sequences tested previously. Three additional sequences were selected based on their relative rarity among most pathogens, and high frequency in the human genome (Fig. 3B). CpG-ODN-3′FITC corresponding to the 31 selected sequences were synthesized.

KIR3DL2^+^NKL cells and KIR^−^NKL cells were cultured separately with each of the thirty-one CpG-ODN-3′FITC. We compared the genomic frequency of these 31 sequences with the extent of their uptake by KIR3DL2^+^NKL cells. Notably, the three oligonucleotides chosen for their prevalence in the human genome, were among those that were poorly taken up by KIR3DL2^+^NKL cells (Fig 3B).

We next explored whether fundamental chemical properties of the 46 oligonucleotides could explain the pattern of oligonucleotide uptake by KIR3DL2^+^NKL cells. No significant correlation was found between KIR3DL2^+^NKL cell uptake, and either the molecular weight, C/G content or the melting temperature of any of the forty-six oligonucleotides (Data S4A–S4C).

To control for the aversion of KIR3DL2^+^NKL cells to human DNA sequences, we divided the relative frequency of each CpG^+^ octamer in a given pathogenic group, by its relative frequency in the human genome (Fig. 3B). This process produced a set of human-normalized genomic CpG^+^ octamer frequencies for each pathogen dataset (Data S5). Overall, the uptake of the 31 CpG^+^ octamers did not directly correlate with the frequencies of those octamers in any dataset of pathogen genomes, either with or without their frequencies being normalized to the human genome (Fig. 3B).

After treatment with IL-12, KIR3DL2^+^ NK cells that bind DNA can transfer it to TLR-9 (Sivori et al., 2010). To see if IL-12 affects the bias of KIR3DL2^+^NKL cells for particular octamer sequences, we cultured KIR3DL2^+^ NK cells for 48 h in medium with IL-2 or the combination of IL-2 and IL-12. Aliquots of these cells were then separately cultured for 4 h with each of the 31 CpG-ODN-3′FITC. Flow cytometry was then used to correlate the uptake of CpG-ODN-3′FITC by cells cultured in medium, either in the presence or absence of IL-12. The correlation was significantly positive with an r^2^ of 0.46 (Data S4D). Cells treated with IL-12 had a similar distribution of gMFI’s as cells not treated with IL-12 (Data S4E). The CpG-ODN-3′FITC having an above average uptake in the absence of IL-12, had a further increase in uptake by the IL-12 stimulated KIR3DL2^+^NKL cells. These results suggest that IL-12 stimulation quantitatively increases the bias of KIR3DL2^+^NKL cells toward specific nucleotide sequences, but does not increase the diversity of nucleotide sequences targeted.

During the course of an 18 h culture, we determined the extent to which each of the 46 different CpG-ODN-3′FITC’s are taken up by KIR3DL2^+^NKL cells. We then compared the uptake of each CpG^+^ octamer with its abundance in the human genome. A striking and statistically significant negative correlation (*p* < 0.0001 and r^2^ = 0.56) was observed (Fig. 3C). This result is consistent with a mechanism in which KIR3DL2^+^NKL cells respond to CpG^+^ DNA sequences that are rare or absent from human genomes, but are tolerant of CpG^+^ DNA sequences that are abundant in human genomes.

Versions of the 46 CpG-ODN that lacked 3′FITC were cultured for 18 h with KIR3DL2^+^NKL cells in medium containing GelRed (see “Materials and Methods”). Analysis of the cells by flow cytometry revealed an impressive negative correlation between the gMFI of GelRed on live KIR3DL2^+^NKL cells, and the relative frequency of the CpG-ODN in the human genome. The statistical significance was found to be *p* < 0.0001 and the r^2^ of the correlation was found to be 0.49 (Fig. 3D). These results support the interpretation that common CpG^+^ sequences in human genomes are poorly taken up by KIR3DL2^+^NKL cells.

### Common CpG^+^ DNA sequences in parasitic worm genomes are taken up abundantly by KIR3DL2^+^NKL cells

We assessed if KIR3DL2 preferentially binds DNA sequences that are specific to a particular group of human pathogens. The uptake of thirty-one CpG-ODN-3′FITC by KIR3DL2^+^NKL cells, was compared with the frequencies of the sequences in HB, HF, HW and HV datasets, as well as in protozoan parasites. Correlations to all of the concatenated datasets were negative, statistically insignificant, and poorly fitted a straight line (Table 1, row ‘Group r^2^’).

**Table 1:** Summary of the correlations between CpG^+^ octamer frequencies in the genomes of pathogenic groups and their uptake by KIR3DL2^+^NKL cells.

	PATHOGEN GENOME FREQUENCY						PATHOGEN GENOME FREQUENCY ÷ HUMAN GENOME FREQUENCY				
	Virus	Fungi	Worm	Bac	Protist		Virus	Fungi	Worm	Bac	Protist
**Group r** ^**2**^	0.0	0.0	0.0	0.0	0.1		0.2	0.1	0.1	0.0	0.1
**Species with r**^**2**^ **> 0.2**	0%	0%	0%	0%	12%		0%	29%	91%	2%	12%
**Best fit of any species (r** ^**2**^ **) **	0.23	0.13	0.16	0.20	0.29		0.22	0.45	0.50	0.29	0.29

We then considered the possibility that a preference for the genetic patterns of a genus or species could be masked by noise arising from the high diversity of species included in the genomic datasets. To address this possibility, we correlated the uptake of thirty-one CpG^+^ODN-3′FITC by KIR3DL2^+^NKL cells with the raw and human-normalized genomic CpG^+^ octamer frequencies for each species in our genomic datasets. A total of 705 species and viral strains was examined.

When examined as a whole, the genomic dataset of parasitic worms was poorly correlated with DNA uptake by KIR3DL2^+^NKL cells, as was the case for all the pathogen datasets. However, when 42 species of parasitic worm were examined individually, 91% (38 species) of the parasitic worms that are relevant to human health correlated with DNA uptake by KIR3DL2^+^NKL cells, having an r^2^ value greater or equal to 0.2. By contrast, only 2% of viral and bacterial species had positive correlations with an r^2^ greater than or equal to 0.2 (Table 1). This indicates that DNA of parasitic worms is preferentially taken up by KIR3DL2^+^NKL cells.

Among those parasitic worms whose genomes that did not correlate with KIR3DL2-DNA uptake, were several species belonging to *Strongyloides* and *Onchocerca*. The CpG octamers in the genomes of these worms have opposing frequencies to the remaining species in the dataset. This explains the lack of correlation between the entire HW dataset and DNA uptake, though 91% of the species in the dataset correlate well individually.

Among all 705 species analyzed, the best fit to the DNA uptake of KIR3DL2^+^NKL cells was the genomic pattern of CpG^+^ octamers in the roundworm *Trichuris trichiura* (r^2^ = 0.50, Bonferroni adjusted *p*-value = 0.01, Table 2). Best fit among fungal species was the common fungus *Aspergillus glaucus* (r^2^ = 0.45), and the slope of this linear regression was also significantly non-zero (Bonferroni adjusted *p*-value = 0.02, Table 2).

**Table 2:** Species from each pathogenic group whose genetically encoded CpG^+^ octamers most closely correlate to the uptake of CpG^+^ octamers by KIR3DL2^+^NKL cells.

Best fit species	Disease	*p*-value	r^2^
*Mycoplasma pneumonia*	Atypical pneumonia	ns	0.29
*Aspergillus glaucus*	Rare infections of nails, ears, and immunocompromised patients	0.02	0.45
*Trichuris trichiura*	Roundworm infection of large intestine	0.01	0.50
*Human herpesvirus 7*	Macular-papular rashes in children	ns	0.22
*Leishmania major*	Cutaneous leishmaniasis.	ns	0.27

**Note:**

Results of linear correlations are shown, with significance of non-zero slope of the line. Bonferroni adjusted *p*-values for 705 tests are reported.

The normalized genomic CpG^+^ octamer frequencies of *Human herpesvirus 7* correlated best with the uptake of DNA by KIR3DL2^+^NKL cells among all human viruses (r^2^ = 0.22). However, when corrected for multiple comparisons, the slope of the linear regression was not significant (Table 2). Among bacterial species, the strongest correlation was with *Mycoplasma pneumonia* (r^2^ = 0.29), which causes atypical pneumonia. However, when corrected for multiple comparisons, the slope of this linear regression was also not significant. Among protozoan species the best fit was *Leishmania major* (r^2^ = 0.27). However, the slope of this line was negative, and also not statitistically significant. In summary, only individual species of pathogenic fungi or parasitic worms correlated significantly with the the DNA uptake of KIR3DL2^+^NKL cells.

### CpG^+^ octamers poorly taken up by KIR3DL2^+^ cells stay at the cell surface

We next assessed if the poor uptake of particular DNA sequences by KIR3DL2^+^NKL cells is due to poor binding or poor internalization. We compared the W1 octamer, which is common to parasitic worm genomes and abundantly taken up by KIR3DL2^+^NKL cells, to the H1 octamer, which is common to the human genome and poorly taken up by KIR3DL2^+^NKL cells. After culture with CpG-ODN-3′FITC for 4 h, KIR3DL2^+^NKL cells were either treated with trypsin, or left untreated. Trypsin digestion effectively removes KIR3DL2 from the surface of cells, as well as any bound CpG-ODN-3′FITC. Any CpG-ODN-3′FITC already internalized by KIR3DL2^+^NKL cells is unaffected by trypsin digestion. Therefore, the ratio of the FITC signals from trypsin-treated and untreated cells is a measure of DNA internalization.

**Figure 4:**
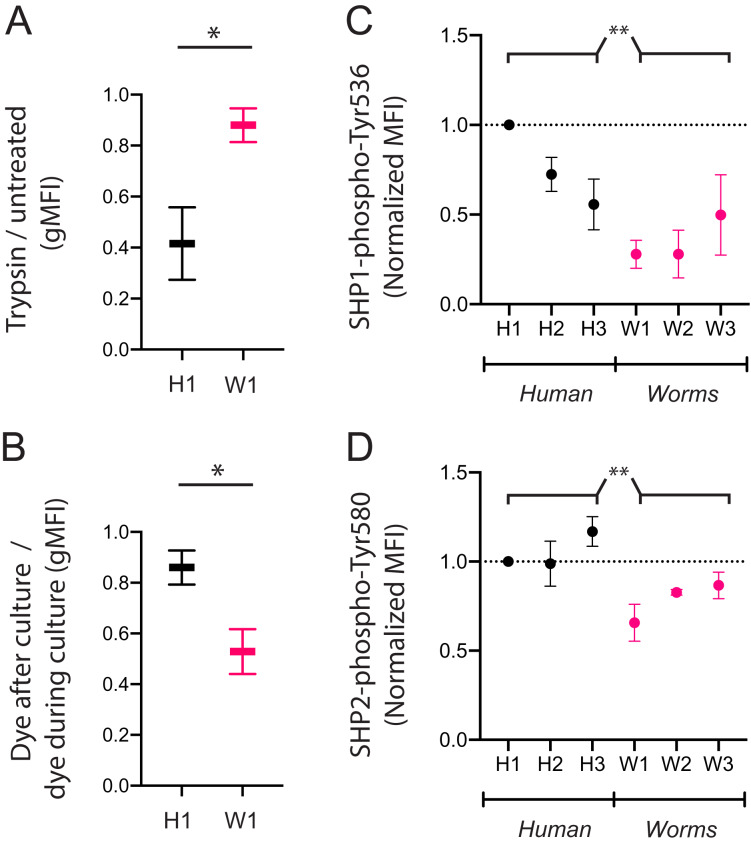
CpG^+^ octamers that are poorly taken up by KIR3DL2^+^ cells remain at the cell surface, where they signal inhibition through SHP-1 and SHP-2. (A) KIR3DL2^+^NKL cells were cultured with the FITC-labeled CpG^+^ H1 or W1 octamer. After 4 hours, cells were either treated with Trypsin, or left untreated. Shown is the ratio of gMFI in the FITC channel of Trypsin-treated cells to untreated cells. Shown are two experiments, and the results of a paired student’s t-test. *p-value = 0.03. (B) KIR3DL2^+^NKL cells were cultured with CpG^+^ octamer H1 or W1, either in the presence or absence of an intercalating DNA dye. After 4 h, dye was added to the cultures that had not already received it. The cells were then analyzed by flow cytometry. Shown is the gMFI ratio of cultures that received dye at the end of culture to those cultures that contained dye throughout the culture period. Shown are two experiments, and the results of a paired student’s t-test. *p-value = 0.04. (C) KIR3DL2^+^NKL cells were cultured for 4 h with CpG^+^ octamers derived either from the human genome (H1, H2, H3) or from the genomes of worms that parasitize humans (W1, W2, W3). Cultures were then fixed, permeabilized, and stained with an antibody targeting SHP1 phosphorylated at Tyr 536. Shown are three experiments, and the results of an ordinary 2-way ANOVA comparing human and worm octamers. **p-value = 0.003. (D) KIR3DL2^+^NKL cells were cultured as in C, but stained with an antibody targeting SHP2 phosphorylated at Tyr 580. Shown are three experiments, and the results of an ordinary 2-way ANOVA comparing human and worm octamers. **p-value = 0.005.

For the KIR3DL2^+^NKL cells cultured with H1, trypsin digestion reduced the FITC signal to 42% of its untreated value (Fig 4A). In contrast, trypsin digestion of KIR3DL2^+^NKL cells cultured with W1 only reduced the FITC signal by 12%. These results are consistent with H1 being bound to KIR3DL2 on the cell surface, but inefficiently internalized.

In confirming these results, we cultured KIR3DL2^+^NKL cells with unlabeled W1 or H1 octamers for 4 h, in medium with or without GelRed. After the 4-h incubation, GelRed was added to those cultures that did not already include it, after which the cells from all cultures were washed immediately. Whereas both bound and internalized DNA were labeled in the cells cultured with dye throughout the 4 hours, only surface-bound DNA was labeled in the KIR3DL2^+^NKL cells that only encountered GelRed at the end of the culture. Thus the ratio of fluorescent signal from cells exposed to dye at the end of culture, and cells dyed throughout culture, is a measure of the proportion of DNA that is present on the cell surface. The ratio of surface DNA to intracellular DNA signal in the KIR3DL2^+^NKL cells cultured with H1 was 0.86 (Fig. 4B) compared to 0.53 for KIR3DL2^+^NKL cells cultured with W1. This result confirms that H1 is bound to KIR3DL2 on the cell surface, but inefficiently internalized.

### DNA sequences that are poorly internalized by KIR3DL2 induce chronic inhibitory signaling

We hypothesized that readily internalized CpG-ODN are likely to cause inhibitory signaling for a short period of time. In contrast, CpG-ODN that bind KIR3DL2, but are not readily internalized, are more likely to lead to chronic inhibition.

To explore these mechanisms, we used a set of CpG-ODN octamers that vary widely in their uptake by KIR3DL2^+^NKL cells and the extent of their internalization. To represent the CpG-ODN that are unlikely to trigger KIR-DNA internalization, we chose the three most frequent CpG-ODN octamers in the human genome: H1, H2 and H3 (Fig. 3A). To represent the CpG-ODN that are expected to internalize rapidly, we chose W1, W2 and W3 (Fig. 3A), the most frequent CpG-ODN octamers of parasitic worm genomes. KIR3DL2^+^NKL cells were cultured with each of the six CpG-ODN octamers for four hours. Cells were then fixed, permeabilized, and stained with either anti-SHP-1(pY536) or anti-SHP-2(pY580) antibodies. These antibodies are specific for the active conformations of their targeted inhibitory proteins.

Addition of H1 induced inhibitory signaling, compared to cells cultured without CpG-ODN (Data S4F). Across repeated experiments, fluorescent signals were normalized to that of live cells cultured with H1. The mean fluorescence intensity (MFI) of anti-SHP-1(pY536) antibody on cells cultured with W1, W2, or W3 was 46% of that obtained by cells cultured with H1, H2 or H3 (Fig. 4C). The MFI of anti-SHP-2(pY580) antibody on cells cultured with W1, W2 or W3 was 74% of that for cells cultured with H1, H2, and H3 (Fig. 4D). The inhibitory signaling of SHP-1 induced by W1, the most abundant CpG^+^ octamer of parasitic worms, was 28% of that induced by H1, the most abundant CpG^+^ octamer in the human genome. These results are consistent with a model in which chronic inhibitory signaling is induced by CpG^+^ octamers that bind to KIR3DL2, but are inefficiently internalized.

### Common CpG^+^ octamers in the human genome reduce the cytolytic and inflammatory responses of KIR3DL2^+^NKL cells to missing-self

We hypothesized that co-culture of KIR3DL2^+^NKL cells with CpG-ODN that are inefficiently internalized and common in human genomes, should diminish cytolytic and inflammatory responses to missing self HLA class I.

**Figure 5:**
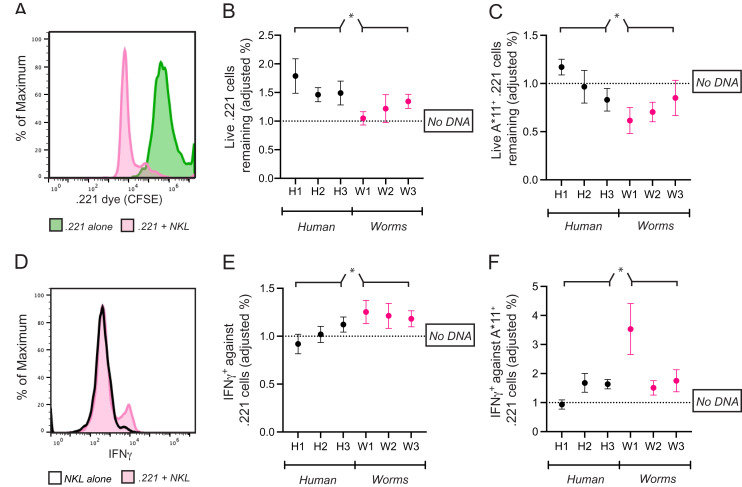
Common CpG^+^ octamers in the human genome reduce cytolysis and IFN-γ production by KIR3DL2^+^NKL cells. (A) Representative FACS plot of CFSE-labeled 721.221 cells in the live gate either alone (green) or in co-culture with KIR3DL2^+^NKL cells for 2.5 h (pink). (B) KIR3DL2^+^NKL cells were cultured with CpG^+^ octamers that are common in the human genome (H1, H2, H3), or the genomes of worms that parasitize humans (W1, W2, W3), or were cultured without exogenous DNA. After four hours, CFSE-labeled 721.221 cells, which lack HLA class I, were added at a 10:1 E:T ratio. Shown is the percentage of live 721.221 cells remaining after 2.5 h of co-culture, divided by the percentage of cells remaining in cultures in which the KIR3DL2^+^NKL cells were not exposed to CpG^+^ octamers. Shown are the results of five experiments, and a 2-way ANOVA comparing human and worm octamers. *p-value = 0.04. (C) The same experimental setup as in B, but with HLA-A*11:01+ 721.221 cells. Shown are the results of three experiments, and a 2-way ANOVA comparing human and worm octamers. *p-value = 0.02. (D) Representative FACS plot of IFNγ in live KIR3DL2^+^NKL cells either cultured alone (black outline), or in co-culture with HLA-A*11:01+ 721.221 cells (pink). (E) KIR3DL2^+^NKL cells were cultured with or without CpG^+^ octamers for 4 h as described in A. HLA^−^ 721.221 cells were then added to the cultures at a 10:1 E:T ratio with BFA. Shown is the percentage of live IFNγ^+^ KIR3DL2^+^NKL cells 6 h later, divided by the percentage of those in co-cultures that were not cultured with CpG^+^ octamers. Shown are the results of seven experiments, and a 2-way ANOVA comparing human and worm octamers. *p-value = 0.03. (F) The same experimental setup as in B, but with HLA-A*11:01+ 721.221 cells being added to the KIR3DL2^+^NKL cells at a 10:1 ratio, instead of 721.221 cells. Shown are the results of four experiments, and a 2-way ANOVA comparing human and worm octamers. *p-value = 0.03.

To address this question, KIR3DL2^+^NKL cells were cultured for 4 h either with H1, H2, H3, W1, W2 or W3, or without any DNA. Cells were then co-cultured with CFSE-labeled lymphoblast cell line 721.221 (221 cells, Fig. 5A). Control CFSE-labeled 221 cells were cultured alone. Because 221 cells lack HLA class I, they induce the KIR3DL2^+^NKL cells to make a cytolytic, missing-self response. After 2.5 h, all cultures were stained with Live/dead dye and analyzed by flow cytometry (see “Materials and Methods”).

The fraction of 221 cells that escaped lysis was calculated by dividing the number of surviving 221 cells in each co-culture by the number of 221 cells that survived in culture alone. To isolate the effect of exogenous DNA on cell lysis, the following ratio was calculated. In each co-culture that included DNA, the percent of 221 cells that escaped lysis was divided by the percent that escaped lysis in the co-culture lacking exogenous DNA. A ratio greater than one shows that the exogenous DNA diminished the cytolytic response of KIR3DL2^+^NKL cells to missing-self HLA class I.

For cultures comprising KIR3DL2^+^NKL cells co-cultured with H1, H2, or H3, the average ratio was 1.58 (Fig. 5B). Thus, 58% more 221 cells (~200 more cells) escaped lysis in these cultures compared to co-cultures without exogenous DNA. In contrast, the co-culture of KIR3DL2^+^NKL cells with W1 had almost no effect on the cytolytic response to missing self (ratio = 1.05, Fig. 5B). Overall, KIR3DL2^+^NKL cells that had been exposed to the three most common CpG^+^ octamers in the human genome lysed significantly fewer missing-self targets than those exposed to the three most common octamers in the genomes of parasitic worms (2-way ANOVA, species factor *p*-value = 0.04, Fig. 5B).

We next assessed if IFNγ production during a missing-self response could be modulated by exogenous DNA. KIR3DL2^+^NKL cells were cultured for 4 h with H1, H2, H3, W1, W2 or W3, or without any DNA. The KIR3DL2^+^NKL cells were then co-cultured with 221 cells for 6 h in medium containing Brefeldin-A, which prevents the secretion of cytokines. Co-cultures were stained with Live/Dead dye, fixed, permeabilized, and stained with anti-IFNγ monoclonal antibody (see “Materials and Methods”). The percentage of IFNγ^+^KIR3DL2^+^NKL cells in each co-culture was divided by the percentage of IFNγ^+^KIR3DL2^+^NKL cells in the co-culture that was not exposed to exogenous DNA (Fig. 5D). The resulting ratio assesses the enhancement (>1) or diminishment (<1) of IFNγ production caused by exogenous DNA.

On average, KIR3DL2^+^NKL cells that were exposed to common CpG^+^ octamers of the human genome produced significantly less IFNγ compared to those exposed to common CpG^+^ octamers of parasitic worm genomes (2-way ANOVA, species factor *p*-value = 0.03, Fig. 5E). For cultures that included H1, H2, or H3, the mean ratio of IFNγ^+^KIR3DL2^+^NKL cells was 1.02 (Fig. 5E). In contrast, the ratio of IFNγ^+^KIR3DL2^+^NKL cells was 1.22 for KIRDL2^+^NKL cells exposed to the W1 sequence of parasitic worms (Fig. 5E).

### Common CpG^+^ octamers in the human genome nullify self-recognition and inhibition by KIR3DL2

KIR-DNA binding and internalization both theoretically impede the ability of KIR3DL2 to bind self-HLA, thereby causing self to appear as non-self to KIR3DL2^+^ cells. We reasoned that the primary immunological role of KIR3DL2-DNA binding might therefore target cells that express HLA-A3/11, rather than those unhealthy cells lacking self-HLA expression.

HLA-A*11:01 triggers inhibition when bound to KIR3DL2. DNA-KIR binding that fails to trigger KIR-DNA internalization allows continued inhibitory signaling (Fig. 4). Taken together, these properties predict that all but the weakest KIR-DNA binding will increase the lysis of HLA-A*11^+^ targets by KIR3DL2^+^NKL cells.

A total of 721.221 cells were stably transfected to express HLA-A*11:01 (221-A11 cell line). KIR3DL2^+^NKL cells were cultured for 4 h with H1, H2, H3, W1, W2, W3, or without any DNA. Cells were then co-cultured with CFSE-labeled 221-A11 cells. Separately, CFSE-labeled 221-A11 cells were cultured alone. After 2.5 h, all cultures were stained with Live/dead dye and analyzed by flow cytometry (see “Materials and Methods”).

As in the experiments with 221 cells, the fraction of 221-A11 cells that escaped lysis in each co-culture was calculated first using those 221-A11 cells that were cultured alone. To isolate the effect of exogenous DNA, a ratio was calculated using the performance of the KIR3DL2^+^NKL cells lacking exogenous DNA as the common denominator. A ratio less than one shows that the exogenous DNA enhanced the cytolytic response of KIR3DL2^+^NKL cells toward HLA^+^ targets.

For cultures comprising KIR3DL2^+^NKL cells co-cultured with H1, H2, or H3, the average ratio of the 221-A11 cells remaining in culture was 0.99. This ratio indicates that common CpG^+^ octamers in the human genome inhibit KIR3DL2^+^NKL cells to a similar extent as HLA-A*11:01. In contrast, a ratio of 0.72 was observed for KIR3DL2^+^NKL cells cultured with W1. This ratio indicates that KIR3DL2^+^NKL cells cultured with CpG^+^ octamers from parasitic worm genomes lyse more HLA-A*11:01^+^ cells than those cultured with CpG^+^ octamers from the human genome (2-way ANOVA, species factor *p*-value = 0.02).

We next tested if the production of IFNγ in response to targets expressing HLA-A*11:01 is modulated by exogenous DNA. KIR3DL2^+^NKL cells were cultured for 4 h with either H1, H2, H3, W1, W2, W3, or without DNA. KIR3DL2^+^NKL cells were then co-cultured with 221-A11 cells for 6 h in medium with Brefeldin-A. Co-cultures were stained with Live/Dead dye, fixed, permeabilized, and then stained with a IFNγ-specific monoclonal antibody (see “Materials and Methods”). The percentage of IFNγ^+^KIR3DL2^+^NKL cells in each co-culture was divided by the percentage of IFNγ^+^KIR3DL2^+^NKL cells that were not exposed to exogenous DNA. The resulting ratio assesses the enhancement (>1) or diminishment (<1) of IFNγ caused by exogenous DNA.

For the KIR3DL2^+^NKL cells cultured with W1, the ratio of IFNγ^+^KIR3DL2^+^NKL cells was 3.53. KIR3DL2^+^NKL cells exposed to W1 produced more than three times the amount of IFNγ, compared to cells not exposed to exogenous DNA. For KIR3DL2^+^NKL cells cultured with H1, the average ratio of IFNγ^+^KIRDL2^+^NKL cells was 0.94.

Overall, these results indicate that the inhibition resulting from self-recognition through KIR3DL2 can be prevented by exposure to oligonucleotide sequences that are frequent in the genomes of parasitic worms.

## Discussion

Experiments in which NK cells were stimulated with whole genomic DNA showed that bacterial DNA stimulates NK cells, whereas mammalian DNA does not (Yamamoto et al., 1992). This difference correlates with the high frequency of CpG motifs in bacterial genomes, and their low frequency in mammalian genomes (Bird, 1980). To our knowledge, our study provides the first demonstration of an NK cell subset that is inhibited by exogenous DNA, depending on the nucleotides flanking the CpG motif. This observation suggests that subsets in the KIR^+^ NK cell repertoire exhibit a range of possible responses to exogenous DNA, including inhibition, and that the responses vary with the DNA sequence.

KIR3DL2 can pass DNA to TLR-9 upon internalization (Sivori et al., 2010). However, TLR-9 requires two CpG motifs on an oligonucleotide that is at least 12 nucleotides in length (Verthelyi et al., 2001), though specific 5-mers can induce immune stimulation when delivered with microspheres (Fearon et al., 2003). That shorter CpG-ODN more readily trigger uptake and internalization by KIR3DL2, may imply that TLR-9 cooperation is not the only object of KIR3DL2-DNA binding. On the other hand, CpG-ODN shorter than those required to activate TLR-9, can augment its activity and compensate for the lack of a second CpG motif (Pohar et al., 2017). Uptake of CpG-ODN plateaued at lengths greater than seven nucleotides, and again at lengths greater than 14 nucleotides. The edges of these plateaus perhaps indicate the binding pocket and maximum binding surface, respectively. Intriguingly, the second plateau defines lengths ideal for TLR-9 stimulation.

It is difficult to imagine that short ODN alone can trigger cross-linking and inhibitory signaling of KIR3DL2. KIR3DL2 is unique among inhibitory KIR in that it forms a dimer on cell surfaces (Pende et al., 1996). Depending on sequence, short ODN could aid dimerization, thereby enhancing tonic inhibitory signaling. Another possibility is that some ODN alter the specificity of KIR3DL2-HLA binding, such that HLA class I allotypes other than A3/11 effectively bind KIR3DL2. Enhanced binding of the A3/11-negative HLA-A expressed on KIR3DL2^+^NKL cells could therefore explain why KIR3DL2 is retained at the surface in the case of inhibitory ODN, and why the resulting inhibitory signaling rivals that induced by HLA-A*11.

There is broad agreement between our experiments using FITC-labeled ODN and those using GelRed dye, as well as with functional experiments in which the DNA was unlabeled. Though large dye molecules might be expected to alter KIR-DNA binding, dyes that label ssDNA typically attach to the negatively charged phosphate groups of the DNA backbone. The agreement between the two dyes, and the significant differences in uptake we observed due to sequence, are consistent with a model in which binding occurs between KIR3DL2 and the nitrogenous bases of ssDNA.

Our data did not identify a simple consensus sequence pattern for high-affinity KIR3DL2-DNA binding. However, the two nucleotides following the CpG motif appear to be crucial. TGGCGTAC was taken up about twice as much as TGGCGATC (Fig. 3B), although the sequences differ only in nucleotides at the sixth and seventh positions, which are swapped. Similarly, octamer GTTCGACC was taken up the most by KIR3DL2^+^NKL cells, whereas substituting the seventh position with adenine yielded GTTCGAAC, which was taken up by only an average amount. In contrast to the concept that positions six and seven dictate binding, CATCGACC was taken up to a lesser extent than the dominant GTTTCGACC, though in this case only the first two nucleotides differ. Such complexity suggests that multiple binding orientations are possible between ssDNA and KIR3DL2, and demands further study.

Because the nucleotides flanking CpG motifs in parasitic worm genomes are the least similar to those of the human genome, the correlation between KIR3DL2-DNA binding and parasite genomes may not indicate a role for NK cells in parasitic infections. Instead, the apparent affinity of KIR3DL2 for parasitic DNA could be the consequence of avoiding the recognition of self-DNA. However, the correlation between CpG^+^ octamer frequencies in parasitic worm species and DNA uptake by KIR3DL2 was strong even after genomic frequencies were normalized to account for a bias against binding human sequences. Because helminth infections are less prevalent in the populations of industrialized countries (de Silva et al., 2003), and who provide the subjects for most studies, such studies may not have uncovered a role for KIR3DL2^+^NK cells in parasitic immunity.

Among the 705 pathogenic species we examined, the best correlation between KIR3DL2-DNA binding and genomic frequencies of CpG^+^ octamers was for *Trichis Trichuria*, a parasitic intestinal nematode. It is therefore intriguing that infection with *T. Trichuria* is associated with an increased level of soluble HLA in the blood (Brown et al., 1999). Soluble HLA-A3/11 binds strongly to KIR3DL2 in a peptide-dependent manner (Hansasuta et al., 2004), and could thus compete with exogenous DNA for KIR3DL2 binding in *T. Trichuria* infected, HLA-A3/11^+^ individuals.

An infection model similar to *T. Trichuria* is the mouse nematode *Heligmosomoides polygyrus bakeri (Hpb)*. Infection of mice with *Hpb* causes greater intestinal damage if NK cells are either absent or unable to produce IFNγ. However, in this case NK cells have no effect on the longevity or viability of the parasite itself (Gentile et al., 2020). This is one example, among many, in which the mammalian immune system has evolved to cooperate with parasites in limiting self-damage (Rook & Brunet, 2005).

The concept of cooperation with parasites could explain some of the features of KIR-DNA binding, when the impact of NK cells on other immune cells is considered. Firstly, the CpG-DNA of parasitic worms removes KIR3DL2 from the surface of NK cells. Such removal could enable NK cells to lyse HLA-expressing cells more easily. Secondly, amongst all human cells, the immune system’s T cells and neutrophils express some of the highest levels of HLA class I (Boegel et al., 2018). If inhibitory KIR that recognize HLA class I were removed from the surface of NK cells by exogenous DNA, then T cells and neutrophils would suffer the greatest increased risk of attack among all of the cells in the body. Thirdly, NK cells have an established role in curtailing CD8^+^ T cell-mediated immunopathogenicity through cytolysis (Marcenaro, Ferranti & Moretta, 2005; Pallmer et al., 2019). In this context, parasite DNA might recruit KIR3DL2^+^ NK cells to quell an emerging anti-parasite immune response, by encouraging them to lyse HLA replete immune cells.

CpG-ID frequencies have been examined in the human genome and the genomes of other species (Bastos et al., 2011; Paci et al., 2016). Bastos et al. (2011) reported CpG-ID in dinucleotide units, summing two frame-shifted passes through the genome. Paci et al. examined the relative frequencies of CpG-ID distributions on a logarithmic scale. Because our goal was to study CpG-ID’s on a scale relevant to KIR3DL2 binding, we focused on short CpG-ID’s 1–20 nucleotides in length. To our knowledge, ours is the first study to report that CpG-ID frequencies in this range are higher than expected at values conforming to the arithmetic sequence 3x-2 in all forms of life.

Individuals having HLA-A*03 and/or HLA-A*11 have KIR3DL2^+^ NK cells that respond aggressively to cells that lack these HLA-A allotypes. This phenomenon is called education (Anfossi et al., 2006) or licensing (Kim et al., 2005) of NK cells. Recently we described a method to educate KIR3DL1^+^ NK cells *in vitro*, using a cell line that expresses the HLA-B Bw4 motif that binds KIR3DL1 (Pugh et al., 2019). In the current study, the soluble CpG^+^ octamer H1 inhibited KIR3DL2^+^NKL cells to an extent that is comparable to that mediated by cell surface HLA-A*11:01. Future studies will address whether DNA sequences that remain bound to surface KIR could be used to educate NK cells.

## Conclusions

We hypothesized that the inhibitory receptor KIR3DL2 binds and internalizes exogenous DNA in a sequence-specific manner, allowing Natural Killer immune cells expressing KIR3DL2 to distinguish self-DNA from pathogen DNA. In order to address this hypothesis, we surveyed the nucleotides flanking CpG sites in the human genome and the genomes of all human pathogens. Those CpG^+^ octamers that were most abundant in the human genome were taken up the least by KIR3DL2^+^NKL cells, caused inhibitory signaling, and reduced both cytolysis and the production of IFNγ. In contrast, those CpG^+^ octamers that are most abundant in the genomes of parasitic worms enhanced the cytolysis and IFNγ production of KIR3DL2^+^NKL cells. We conclude that KIR3DL2 allows Natural Killer cells to differentiate self-DNA from pathogen DNA.

## Supplemental Information

10.7717/peerj.12258/supp-1 Supplemental Information 1 Data establishing the experimental parameters of CpG-DNA uptake by KIR3DL2^+^NKL cells. (A) FACS plot of NKL cells (grey), KIR3DL2^+^NKL cells (red), and KIR3DL1^+^NKL cells (blue). (B) FACS plot of cell lines as in A, after culture with FITC-labeled Seq1 DNA. (C) Flow cytomtery results of KIR3DL2^+^NKL cells and KIR3DL1^+^NKL cells cultured with FITC-labeled Seq1 DNA for four hours at the indicated concentrations. Representative of at least three separate experiments. (D) Flow cytometry results of KIR3DL2^+^NKL cells cultured with 50mM FITC-labeled Seq1 DNA for two hours at either 37°C or 4°C in plates either treated or not treated for tissue culture. The results of Tukey’s multiple comparison test of 1-way paired ANOVA is shown. ** = p<0.01, *=p<0.05. (E) FACS plot of KIR3DL2^+^NKL cells cultured with a CpG-DNA oligo (red) or GpC-DNA oligo (blue). (F) KIR3DL2^+^NKL cells were cultured with either single-stranded (ss) or double-stranded (ds) CpG-DNA of 10 different lengths. Shown are the results of a paired student’s t-test. ** = p<0.01. (G) Flow cytometry results of KIR3DL2^+^NKL cells cultured with FITC-labeled Seq1 DNA for various durations. Three replicates are shown for each set of timepoints. (H) Flow cytometry results of KIR3DL2^+^NKL cells cultured with one of six FITC-labeled CpG^+^ octamers for times of 4-24 hours. Results are representative of at least four experiments.

10.7717/peerj.12258/supp-2 Supplemental Information 2 Sequences of 3’-FITC-labeled DNA oligonucleotides and variation in uptake of CpG-DNA by CpG-interdistance. (A) Table of 3’-FITC-labeled DNA oligonucleotides with a central CpG motif and lengths ranging from L=5 to L=40 nucleotides. (B) Table of 3’-FITC-labeled DNA oligonucleotides with two, three, or four CpG motifs of lengths ranging from L=10 to L=40 nucleotides. (C) Table of 3’-FITC-labeled oligonucleotides with CpG interdistances of 1-8 bases. (D) Table of 3’-FITC-labeled adducts composed of core sequences (1x) repeated one, two, or three times (2x, 3x, 4x). (E) Flow cytometry results of KIR3DL2^+^NKL cells cultured with FITC-labeled oligonucleotides listed in C. Shown are results of three experiments. The gMFI in each experiment is normalized to the signal obtained by the CpG-Interdistance = 1.

10.7717/peerj.12258/supp-3 Supplemental Information 3 CpG-interdistances of broad genomic datasets, distribution of nucleotides flanking CpG motifs in ssDNA viruses by host specificity, and the twenty most frequent CpG^+^ octamers in each genomic dataset. (A) The frequency of CpG interdistances in datasets of bacteria *B*, fungi *F*, viruses *V*, worms *W* and the human genome *H*. Frequencies were calculated by dividing the count of each interdistance by the total number of bases in the dataset. (B) The top 20 most frequently encoded CpG octamers in genomic datasets. Datasets include those listed in A and genomic datasets of human pathogens including human bacteria *HB*, human fungi *HF*, human viruses *HV* and human worms *HW*. C, Distribution of bases encoded in octamers with a central CpG site in each genomic dataset. The distribution of CpG^+^ octamers in the human genome was compared to the distribution in each non-human dataset using the Pearson correlation coefficient.

10.7717/peerj.12258/supp-4 Supplemental Information 4 The uptake of CpG^+^ octamers by KIR3DL2^+^NKL cells does not correlate with molecular constants, and is not skewed by IL-12 stimulation. (A) (y-axis) uptake of 31 CpG+ODN-3’FITC octmaers and (x-axis) the molecular weight of the octamers. (B) As in A, but the x-axis gives the C/G content of the octamers. (C) As in A, but the x-axis gives the melting temperature of the octamers. (D) KIR3DL2^+^NKL cells were cultured for 48 hours in medium with IL-2 and IL-12, or IL-2 alone. Cells were then cultured with the 31 octamers for four hours, and then analyzed by flow cytometry. Shown is the gMFI in the FITC channel of cells in the live gate. (E) Data in D, presented in x-y format. Shown is the simple linear regression line, with analysis of the goodness-of-fit and significance of the slope. (F) Flow cytometry histogram of anti-SHP-1(pY536) signal on KIR3DL2^+^NKL cells cultured with H1 CpG-ODN for 20 minutes (blue), compared to cells not cultured with exogenous DNA (red).

10.7717/peerj.12258/supp-5 Supplemental Information 5 Genomic data used in this study, results of CpG octamer counts in each genomic dataset, and the data comprising the main figures. (Tab 1) Genomic data references, descriptions, and the datasets in which they were included. (Tab 2) Raw and adjusted data for CpG octamers in each genomic dataset. (Tabs 3-7) Raw data comprising each main figure.

## Additional Information and Declarations

### Data Availability

The following information was supplied regarding data availability: Data S5 includes raw data and a list of all genomic sequences used, which are all publicly available. The computer code is available at GitHub: https://github.com/jpugh-sudo/Pugh_SF_6.
