# Supplementary figures and images for: Abundant CpG-sequences in human genomes inhibit KIR3DL2-expressing NK cells
Source: PeerJ. 2021 Nov 5;9:e12258. doi: 10.7717/peerj.12258 (PMC8574216; doi:10.7717/peerj.12258)

# Supplemental Data 1

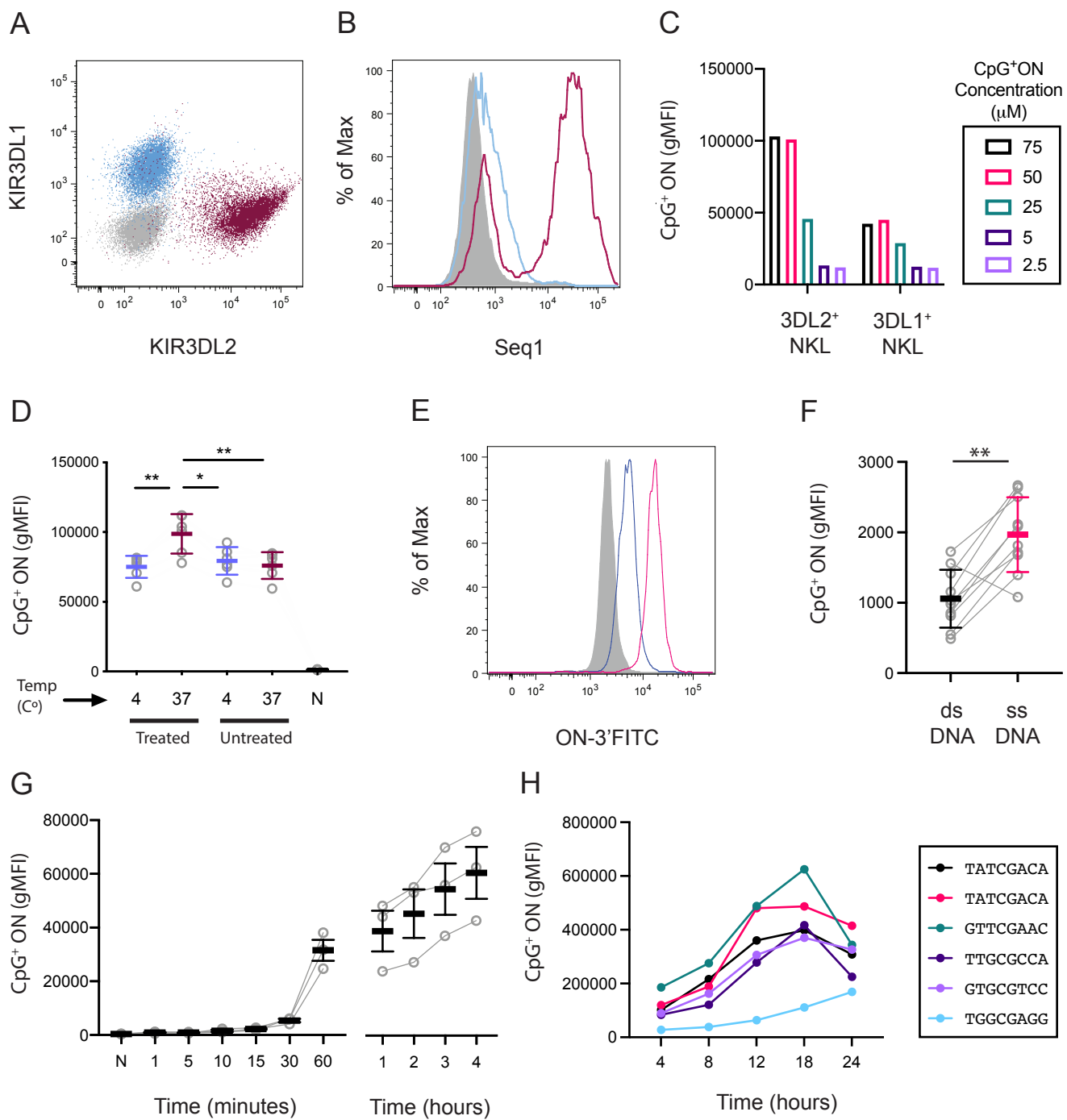

Supplement: Supplemental Information 1 — (A) FACS plot of NKL cells (grey), KIR3DL2+NKL cells (red), and KIR3DL1+NKL cells (blue). (B) FACS plot of cell lines as in A, after culture with FITC-labeled Seq1 DNA. (C) Flow cytomtery results of KIR3DL2+NKL cells and KIR3DL1+NKL cells cultured with FITC-labeled Seq1 DNA for four hours at the indicated concentrations. Representative of at least three separate experiments. (D) Flow cytometry results of KIR3DL2+NKL cells cultured with 50mM FITC-labeled Seq1 DNA for two hours at either 37°C or 4°C in plates either treated or not treated for tissue culture. The results of Tukey’s multiple comparison test of 1-way paired ANOVA is shown. ** = p<0.01, *=p<0.05. (E) FACS plot of KIR3DL2+NKL cells cultured with a CpG-DNA oligo (red) or GpC-DNA oligo (blue). (F) KIR3DL2+NKL cells were cultured with either single-stranded (ss) or double-stranded (ds) CpG-DNA of 10 different lengths. Shown are the results of a paired student’s t-test. ** = p<0.01. (G) Flow cytometry results of KIR3DL2+NKL cells cultured with FITC-labeled Seq1 DNA for various durations. Three replicates are shown for each set of timepoints. (H) Flow cytometry results of KIR3DL2+NKL cells cultured with one of six FITC-labeled CpG+ octamers for times of 4-24 hours. Results are representative of at least four experiments. [file peerj-09-12258-s001.pdf]

A

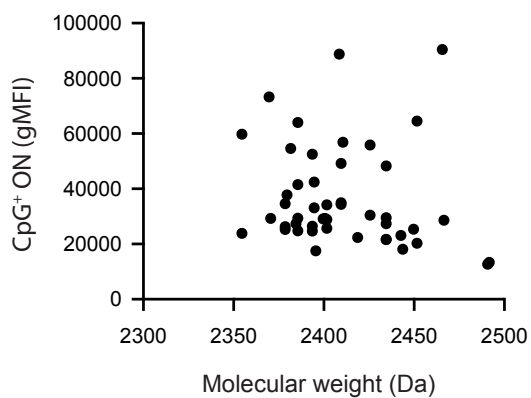

B

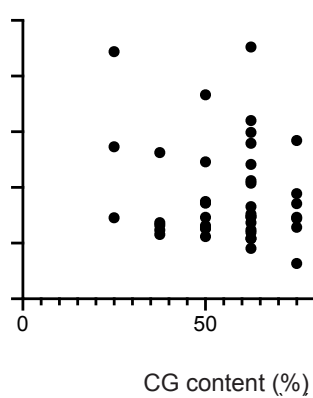

C

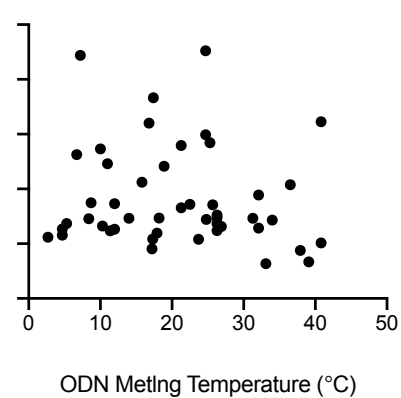

D

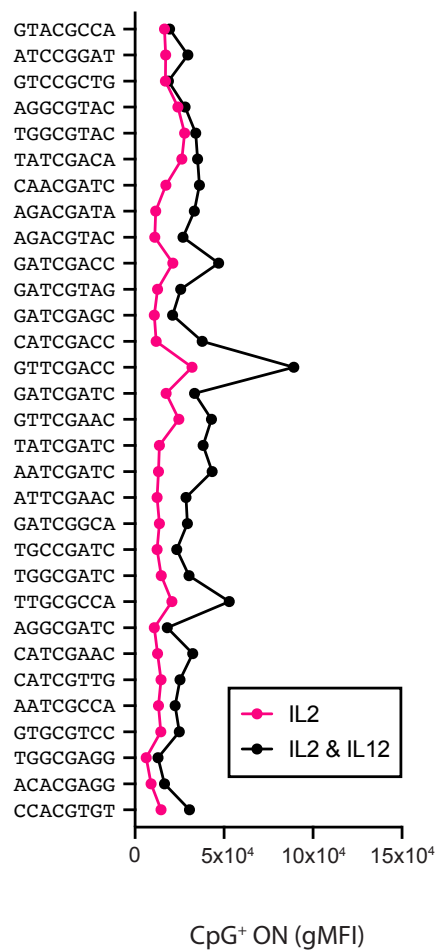

E

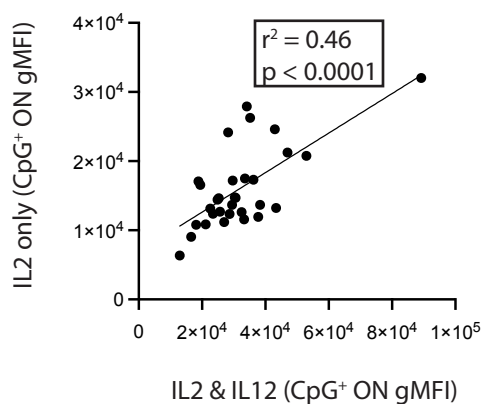

F

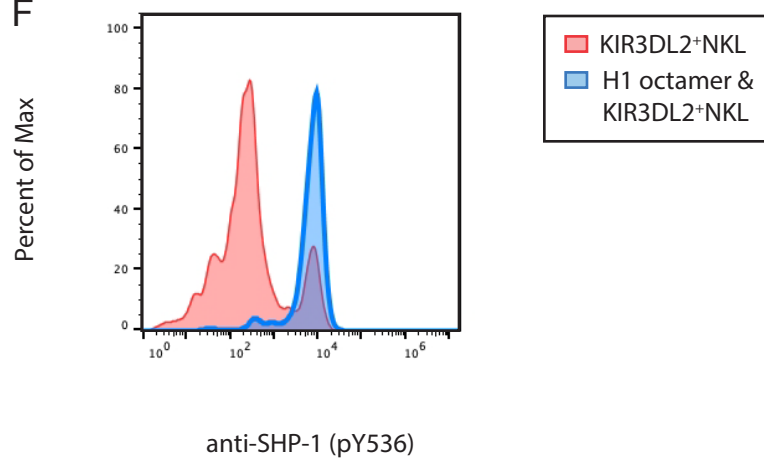

Supplement: Supplemental Information 4 — (A) (y-axis) uptake of 31 CpG+ODN-3’FITC octmaers and (x-axis) the molecular weight of the octamers. (B) As in A, but the x-axis gives the C/G content of the octamers. (C) As in A, but the x-axis gives the melting temperature of the octamers. (D) KIR3DL2+NKL cells were cultured for 48 hours in medium with IL-2 and IL-12, or IL-2 alone. Cells were then cultured with the 31 octamers for four hours, and then analyzed by flow cytometry. Shown is the gMFI in the FITC channel of cells in the live gate. (E) Data in D, presented in x-y format. Shown is the simple linear regression line, with analysis of the goodness-of-fit and significance of the slope. (F) Flow cytometry histogram of anti-SHP-1(pY536) signal on KIR3DL2+NKL cells cultured with H1 CpG-ODN for 20 minutes (blue), compared to cells not cultured with exogenous DNA (red). [file peerj-09-12258-s004.pdf]
