# Supplementary material for: Abundant CpG-sequences in human genomes inhibit KIR3DL2-expressing NK cells
Source: PeerJ. 2021 Nov 5;9:e12258. doi: 10.7717/peerj.12258 (PMC8574216; doi:10.7717/peerj.12258)
Supplement: Supplemental Information 2 — (A) Table of 3’-FITC-labeled DNA oligonucleotides with a central CpG motif and lengths ranging from L=5 to L=40 nucleotides. (B) Table of 3’-FITC-labeled DNA oligonucleotides with two, three, or four CpG motifs of lengths ranging from L=10 to L=40 nucleotides. (C) Table of 3’-FITC-labeled oligonucleotides with CpG interdistances of 1-8 bases. (D) Table of 3’-FITC-labeled adducts composed of core sequences (1x) repeated one, two, or three times (2x, 3x, 4x). (E) Flow cytometry results of KIR3DL2+NKL cells cultured with FITC-labeled oligonucleotides listed in C. Shown are results of three experiments. The gMFI in each experiment is normalized to the signal obtained by the CpG-Interdistance = 1. [file peerj-09-12258-s002.pdf]

Supplemental Data 2

A

Length (Bases)	Sequence
L=5	TTCGTF
L=6	TTCGTTF
L=7	TTTCGTTF
L=8	TTTCGTTTF
L=9	TTTTCGTTTF
L=10	TTTTCGTTTTF
L=12	TTTTTCGTTTTTF
L=14	TTTTTTCGTTTTTTF
L=16	TTTTTTTCGTTTTTTTF
L=18	TTTTTTTTCGTTTTTTTTF
L=21	TTTTTTTTTTCGTTTTTTTTTF
L=24	TTTTTTTTTTTCGTTTTTTTTTTTF
L=28	TTTTTTTTTTTTTCGTTTTTTTTTTTTTF
L=30	TTTTTTTTTTTTTTCGTTTTTTTTTTTTTTF
L=32	TTTTTTTTTTTTTTTCGTTTTTTTTTTTTTTTF
L=35	TTTTTTTTTTTTTTTTTCGTTTTTTTTTTTTTTTTF
L=40	TTTTTTTTTTTTTTTTTTTCGTTTTTTTTTTTTTTTTTTTF

B

Length (Bases)	CpG motifs	Sequence
L=10	2 x CpG	TTCGTTTCGTF
L=12	2 x CpG	TTCGTTTTCGTTF
L=14	2 x CpG	TTCGTTTTTCGTTTF
L=15	2 x CpG	TTTCGTTTTTTCGTTTF
L=18	2 x CpG	TTTCGTTTTTTTCGTTTTF
L=21	3 x CpG	TTCGTTTTTCGTTTTTCGTTTF
L=24	4 x CpG	TTCGTTTTCGTTTTCGTTTTCGTTF
L=30	3 x CpG	TTTTCGTTTTTTTTCGTTTTTTTTCGTTTTF
L=36	4 x CpG	TTTCGTTTTTTCGTTTTTTCGTTTTTTCGTTTF
L=40	4 x CpG	TTTTCGTTTTTTTTCGTTTTTTTTCGTTTTTTTTCGTTTTF

C

CpG Interdistance	Sequence
CpG-ID = 1	TTTTCGTCGTTTTTF
CpG-ID = 2	TTTTCGTTCGTTTTF
CpG-ID = 3	TTTCGTTTCGTTTTF
CpG-ID = 4	TTTCGTTTTCGTTTF
CpG-ID = 5	TTCGTTTTTCGTTTF
CpG-ID = 6	TTCGTTTTTTCGTTF
CpG-ID = 7	TCGTTTTTTTCGTTF
CpG-ID = 8	TCGTTTTTTTTCGTF

E

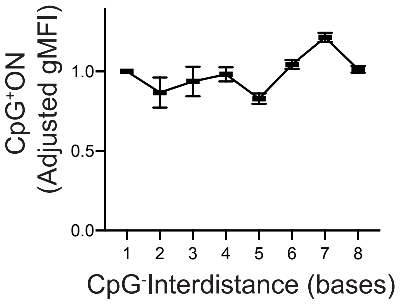

D

Core Length	Core Sequence	Repeats	Full Sequence
Pentamer	TTCGT	1x	TTCGTF
		2x	TTCGTTTCGTF
		3x	TTCGTTTCGTTTCGTF
		4x	TTCGTTTCGTTTCGTTTCGTF
Hexamer	TTCGTT	1x	TTCGTTF
		2x	TTCGTTTTCGTTF
		3x	TTCGTTTTCGTTTTCGTTF
		4x	TTCGTTTTCGTTTTCGTTTTCGTTF
Heptamer	TTTCGTT	1x	TTTCGTTF
		2x	TTTCGTTTTTCGTTF
		3x	TTTCGTTTTTCGTTTTTCGTTF
		4x	TTTCGTTTTTCGTTTTTCGTTTTTCGTTF
Octoamer	TTTCGTTT	1x	TTTCGTTTF
		2x	TTTCGTTTTTTCGTTTF
		3x	TTTCGTTTTTTCGTTTTTTCGTTTF
		4x	TTTCGTTTTTTCGTTTTTTCGTTTTTTCGTTTF
Nonamer	TTTTCGTTT	1x	TTTTCGTTTF
		2x	TTTTCGTTTTTTTCGTTTF
		3x	TTTTCGTTTTTTTCGTTTTTTTCGTTTF
		4x	TTTTCGTTTTTTTCGTTTTTTTCGTTTTTTTCGTTTF
Decamer	TTTTCGTTTT	1x	TTTTCGTTTTF
		2x	TTTTCGTTTTTTTTCGTTTTF
		3x	TTTTCGTTTTTTTTCGTTTTTTTTCGTTTTF
		4x	TTTTCGTTTTTTTTCGTTTTTTTTCGTTTTTTTTCGTTTTF
