# Supplementary material for: Abundant CpG-sequences in human genomes inhibit KIR3DL2-expressing NK cells
Source: PeerJ. 2021 Nov 5;9:e12258. doi: 10.7717/peerj.12258 (PMC8574216; doi:10.7717/peerj.12258)
Supplement: Supplemental Information 3 — (A) The frequency of CpG interdistances in datasets of bacteria B, fungi F, viruses V, worms W and the human genome H. Frequencies were calculated by dividing the count of each interdistance by the total number of bases in the dataset. (B) The top 20 most frequently encoded CpG octamers in genomic datasets. Datasets include those listed in A and genomic datasets of human pathogens including human bacteria HB, human fungi HF, human viruses HV and human worms HW. C, Distribution of bases encoded in octamers with a central CpG site in each genomic dataset. The distribution of CpG+ octamers in the human genome was compared to the distribution in each non-human dataset using the Pearson correlation coefficient. [file peerj-09-12258-s003.pdf]

Supplemental Data 3

A

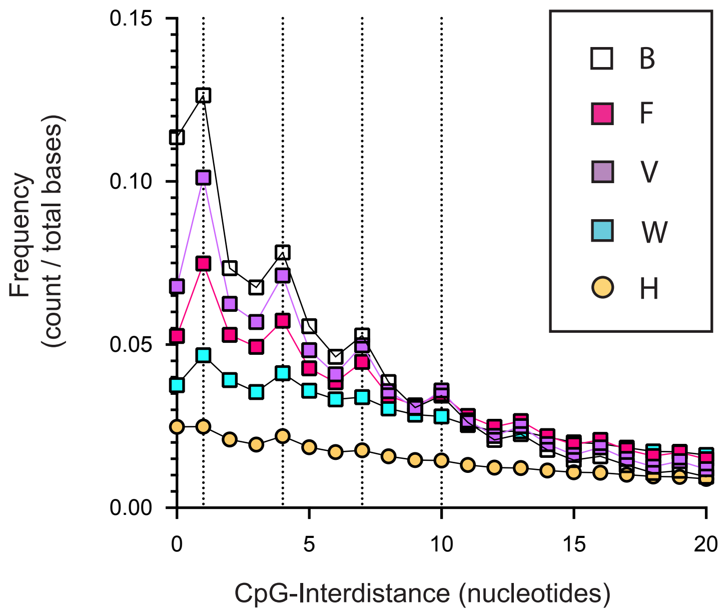

C

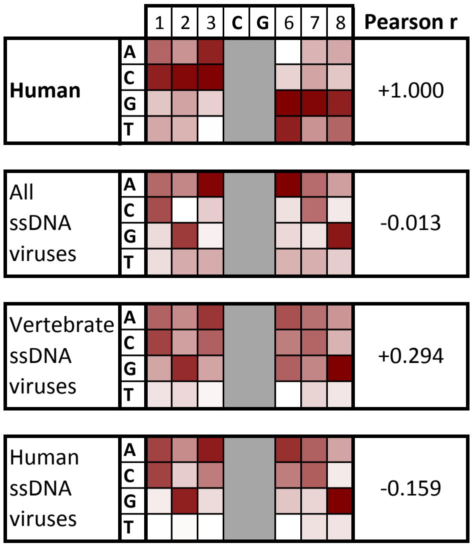

B

Rank	Human	B	F	V	W	HB	HF	HV	HW
1	CCCCGCCC	CGCCGCCG	CATCGTCA	CGCCGCCG	TTTCGAAA	CGCCGCCG	CATCGTCA	GCGCGCGC	TTTCGAAA
2	GGGCGGGG	CGGCGGCG	TGACGATG	CGGCGGCG	AAACGAAA	CGGCGGCG	TGACGATG	GCGCGCGA	AAACGAAA
3	CCACGTGG	CGCCGCGC	CATCGACA	CGACGACG	TTTCGTTT	CGCCGCGC	CATCGCCA	CGGCGGCC	TTTCGTTT
4	CCCCGGCC	GCGCGGCG	TGTCGATG	CGTCGTCG	ATTCGAAA	GCGCGGCG	TGGCGATG	CGCCGCCG	AAACGTTT
5	GGCCGGGG	CGGCGCCG	CATCGCCA	CGACGCCG	TTTCGAAT	CGGCGCGC	CATCGACA	CGCCGCGC	ATTCGAAA
6	CCCCGGGG	CGGCGCGC	TGGCGATG	CGGCGTCG	AATCGAAA	GCGCGCCG	TGTCGATG	CGCCGCCC	TTTCGAAT
7	AAACGTTT	GCGCGCCG	CATCGTCG	CGACGGCG	TTTCGATT	CGGCGCCG	CATCGTCG	GCGCGCCC	AAACGAAT
8	GCCCGGCC	CGCCGGCG	CGACGATG	CGCCGTCG	AAACGTTT	CGCCGGCG	CGACGATG	GCGCGGCC	ATTCGTTT
9	GGCCGGGC	CCGCGCCG	CAACGCCA	CGCCGACG	ATTCGAAT	CCGCGCCG	CAACGCCA	GCGCGCGG	AATCGAAA
10	AGGCGGGG	CGGCGCGG	TGGCGTTG	CGTCGGCG	AATCGATT	CGGCGCGG	TGGCGTTG	CGGCGCGC	TTTCGATT
11	CCCCGCCT	CGGCGGCC	CAACGACA	CGGCGACG	AAACGAAT	CGGCGGCC	CAACGACA	CGGCGGCG	ATTCGAAT
12	CCCCGGGC	GGCCGCCG	TGTCGTTG	CGTCGCCG	ATTCGTTT	GGCCGCCG	TGTCGTTG	CCGCGCGC	AAGCGAAA
13	GCCCGGGG	CGCCGACG	CGACGAGA	CGCCGCGC	AATCGAAT	GCGCGCGC	CATCGATG	CCGCGGCC	TTTCGCTT
14	CAGCGTGG	CGTCGGCG	TCTCGTCG	GCGCGGCG	ATTCGATT	CGCCGACG	CTTCGTCA	CGCCGCCA	AAACGATT
15	CCACGCTG	CGACGCCG	CACCGCCA	CGGCGCCG	AATCGATA	CGTCGGCG	TGACGAAG	GGCCGCGC	AATCGTTT
16	CCCCGCAG	CGGCGTCG	TGGCGGTG	CGGCGCGC	TATCGATT	CGGCGGCA	CTTCGAGA	CCCCGCCC	GAACGAAA
17	CTGCGGGG	CGGCGACG	CCTCGTCG	GCGCGCCG	AAACGATT	TGCCGCCG	TCTCGAAG	GCGCGGCG	TTTCGTTC
18	CCCCGAGG	CGTCGCCG	CGACGAGG	CCGCGCCG	AATCGTTT	CGCCGCCA	CAGCGCCA	CGCCGCGA	CAACGAAA
19	CCTCGGGG	CGACGGCG	CGACGAAG	CGGCGCGG	AAGCGAAA	TGGCGGCG	TGGCGCTG	TGGCGGCG	TTTCGTTG
20	AAACGAAA	CGCCGTCG	CTTCGTCG	CGCCGCCA	TTTCGCTT	CGGCGACG	CGACGAAG	GCGCGGGC	AATCGAAT
